# Purification of SlREC2 from wild-type tomato leaflets using immunoaffinity chromatography and immunoprecipitation

**DOI:** 10.3389/fpls.2025.1616665

**Published:** 2025-12-17

**Authors:** Lingling Zhu, Robert M. Larkin

**Affiliations:** National Key Laboratory for Germplasm Innovation and Utilization of Horticultural Crops, College of Horticulture and Forestry Sciences, Huazhong Agricultural University, Wuhan, China

**Keywords:** multi subunit protein complexes, protein purification, immunoaffinity chromatography, immunoprecipitation, reduced chloroplast coverage, SlREC2

## Abstract

The process of obtaining greater mechanistic insight into important traits that follows the linking of a gene to a trait can be impeded if the encoded protein has no known biochemical function or a vague biochemical function. In such instances, many researchers attempt to obtain insight into biochemical function by identifying other proteins that bind their protein of interest (POI). Particular methods may lead to the discovery of protein-protein interactions that are not physiologically meaningful. Other methods require the production of transgenic plants, which is impractical or impossible for many important crop plants. We present guidelines for purifying native proteins from wild-type plants because native proteins may stably associate with physiologically meaningful POI-binding proteins (POI-BPs). This strategy involves partially purifying native multisubunit protein complexes from wild-type plants and ultimately purifying native multisubunit protein complexes using immunoaffinity chromatography or immunoprecipitation with affinity-purified polyclonal antibodies raised against a recombinant POI. Proteins that copurify with a POI are candidate POI-BPs that can be identified using mass spectrometry. As an illustrative example, we developed polyclonal antisera against the REDUCED CHLOROPLAST COVERAGE 2 protein from tomato (*Solanum lycopersicum* L.) (SlREC2) and affinity purified the anti-SlREC2 antibodies. We used these antibodies to detect SlREC2 during the partial purification of SlREC2 using rapid batch binding and step gradient elution procedures involving SP Sepharose and Q Sepharose and to purify SlREC2 ultimately using immunoaffinity chromatography and in a distinct procedure using immunoprecipitation. The purified native SlREC2 was readily detectable in Coomassie blue-stained SDS gels and was unambiguously identified using mass spectrometry. We discuss critical parameters and potential technical problems and provide suggestions for developing procedures for purifying a variety of different native POIs with different properties from wild-type plants.

## 1 Introduction

To help improve the quality and yield of crop plants, plant scientists have a critical need for greater mechanistic insight into the important traits. Mechanistic insight is impeded if a gene that is newly linked to an important trait encodes a protein with no known biochemical function or a vague biochemical function. Biochemical functions typically involve binding another molecule to catalyze or to help catalyze a reaction or to contribute to a structure. Currently, 39% of the proteins encoded by the Arabidopsis genome have no known biochemical function (i.e., molecular function) (Reiser et al., 2024). To determine the biochemical function of a protein of interest (POI), many researchers identify other proteins that bind their POI because the POI-binding proteins (POI-BPs) may have a known biochemical function and therefore, may shed light on the biochemical function of the POI. A variety of experimental methods are available for identifying POI-BPs (Elhabashy et al., 2022). Many plant biology researchers rely on heterologous systems, especially two-hybrid methods. Two-hybrid screens are straightforward because they require only the construction of plasmids and the transformation of yeast. Although two-hybrid screens sometimes yield physiologically meaningful POI-BPs, they are useful only for identifying pairwise interactions (Struk et al., 2019). Moreover, even when two-hybrid screens yield physiologically meaningful POI-BPs, they often yield numerous additional POI-BPs that are biophysically genuine but appear to not be physiologically meaningful (Couto et al., 2016; Cheng et al., 2017). Indeed, a major conclusion from years of screening for POI-BPs using two-hybrid methods is that numerous proteins that do not interact in wild-type organisms readily interact in the yeast two-hybrid system (Vidal and Fields, 2014). This problem occurs in plant cells too. For this reason, geneticists insist that the phenotypes induced by gain-of-function alleles reflect the natural activity of a gene only if an opposite phenotype is obtained with a loss-of-function allele of the same gene (Weigel et al., 2000). Therefore, attempting to validate two-hybrid screens with experiments that involve transiently overexpressing two-hybrid interactors in plant cells and testing for protein-protein interactions (e.g., using biomolecular fluorescence complementation assays, luciferase complementation assays, or co-immunoprecipitation of overexpressed proteins) does not distinguish between protein-protein interactions that are physiologically meaningful and protein-protein interactions that are biophysically genuine but not physiologically meaningful. Additionally, two hybrid methods may not detect physiologically relevant POI-BPs for a variety of technical reasons. For example, construction of the fusion protein may obscure the POI-BP binding site or induce an abnormal spatial arrangement between the DNA binding domain and activation domain. The POI may influence transcription, misfold, require posttranslational modifications that do not occur in yeast, become toxic in yeast, or require a specific cellular environment for interactions to occur (Van Criekinge and Beyaert, 1999). An alternative strategy for validating two-hybrid interactors involves testing for phenotypic similarities between mutants deficient in the POI and candidate POI-BPs. This strategy may be time intensive if there is no analogous trait in a model plant that grows rapidly (e.g., Arabidopsis). Moreover, particular phenotypes are common (e.g., mutations in large numbers of different genes lead to chlorophyll deficiency and embryo lethality) and therefore do not provide compelling evidence that a candidate POI-BP is physiologically meaningful. Alternatively, if the POI and POI-BPs are encoded by multigene families, evidence of phenotypic similarities among mutants deficient in POIs and POI-BPs may require the time-intensive construction of higher-order mutants even if a rapidly growing model plant can serve as a suitable experimental system.

Another popular strategy among plant biology researchers is to fuse a POI to one or more epitopes or to another protein (e.g., green fluorescent protein), express the tagged POI in stably transformed plants, extract the POI from plant tissues, and use affinity resins that bind particular tags to purify the tagged protein (Van Leene et al., 2015). Numerous problems can arise from a strategy that depends on transgenic plants, including transgene-induced silencing (Hung and Slotkin, 2021) and the accumulation of POIs at levels that exceed the levels of POI-BPs. When POIs are ectopically expressed or overexpressed, plant cells resemble ligand-binding assays programmed with excessive amounts of a ligand-binding protein (Jarmoskaite et al., 2020) in that the POI will bind all the physiologically meaningful POI-BPs. The remaining POI will be free to participate in biophysically genuine protein-protein interactions that are not physiologically meaningful and therefore to recreate a major problem of two-hybrid methods in plant cells. Even experiments that rely on native promoters to drive the expression of tagged proteins can produce abnormal levels of expression or ectopic expression of POIs because of position effects. Tagged POIs should be expressed in mutants that do not accumulate the POI to avoid a competition for physiologically meaningful POI-BPs between the tagged POI and the native POI. Even if all these issues are resolved and suitable transgenic lines are successfully constructed, epitope and protein tags may disrupt protein-protein interactions required for the assembly of native multisubunit protein complexes. Also, constructing transgenic plants expressing tagged POIs is not practical for researchers working on crops that are difficult to transform (Anjanappa and Gruissem, 2021) or that grow slowly.

Another approach for identifying POI-BPs involves expressing a POI or POI fragment in *Escherichia coli* or some other heterologous system that produces high levels of recombinant protein, purifying the recombinant POI, developing polyclonal antisera against the POI, and using affinity-purified anti-POI antibodies to purify the native POI from wild-type plants, ultimately using immunoaffinity chromatography or immunoprecipitation. This strategy takes advantage of the capacity of wild-type plants to accumulate predominantly physiologically meaningful multisubunit protein complexes and, therefore, is less prone to the artifactual results commonly produced by heterologous systems and by ectopically expressing or overexpressing POIs. Moreover, this approach does not require the production of transgenic plants or the availability of mutants deficient in the POI.

This approach has been used to study protein-protein interactions in nonplant systems (e.g., Dynlacht et al., 1991; LeRoy et al., 1998; Qanungo et al., 2004). Although this method has been used to purify POIs from Arabidopsis twice (Larkin et al., 2003; Bäckström et al., 2007), detailed guidelines for developing methods to purify native proteins from plants would facilitate the broad application of these methods to plant systems. Although a protocol was published for the purification of an Arabidopsis membrane protein, this method may not be broadly applicable. Moreover, the authors did not provide advice on modifying their method for the purification of other proteins with different properties. For example, their POI did not appear to be highly sensitive to proteases and was stable during freezing and thawing (Elmore and Coaker, 2011), which is not true for all proteins (Deutscher, 2009). Additionally, Elmore and Coaker (2011) recommend that researchers identify POI-BPs from complex mixtures generated from immunoprecipitations performed using whole-leaf extracts, which may not lead to success for low-abundance proteins or for proteins that are extremely protease sensitive and therefore become degraded during the typically slow immunopurification step—even in the presence of protease inhibitors. Datasets that are easier to interpret can be obtained by partially purifying native multisubunit protein complexes prior to immunoaffinity purification (Larkin et al., 2003; Bäckström et al., 2007). Immunoaffinity chromatography and immunoprecipitation typically provide 1,000-fold to 10,000-fold purifications (Harlow and Lane, 1999). Therefore, adding some other type of purification prior to the immunoaffinity purification may yield a final fraction with POI-BPs co-purifying with the POI in stoichiometric amounts and at higher levels than proteins that are not POI-BPs but contaminate—in substoichiometric amounts—the final fraction that is eluted from immunoaffinity columns or from immunoprecipitates. Such enrichments are possible even if the native multisubunit complex partially dissociates during the purification (Larkin et al., 2003). Interpretation of data such as these is straightforward. Initial purifications prior to immunoaffinity purification may also separate the native POI from proteases, which may degrade POIs during the typically slow immunoaffinity purification steps. Initial purification steps include precipitation techniques (e.g., ammonium sulfate precipitation), the purification of organelles or organellar fractions, and the fractionation of whole-organ extracts or organellar fractions using ion exchange, affinity, multimodal, or gel filtration chromatography. This approach will also be useful for identifying amino acid residues in POIs with physiologically meaningful posttranslational modifications because this approach allows researchers to avoid problems associated with using transgenic plants described above.

The REDUCED CHLOROPLAST COVERAGE (REC) proteins are encoded by small gene families in plants and play a central role in allocating cellular space to chloroplasts and chromoplasts in Arabidopsis and tomato (*Solanum lycopersicum* L.). The biochemical functions of the REC proteins are unknown and difficult to predict (Larkin et al., 2016; Hu et al., 2024). The amount of cellular space allocated to the plastid compartment in leaves and fruits is reduced the most in *slrec2* mutants relative to wild type (Hu et al., 2024). We developed a method for the rapid purification of the SlREC2 protein from wild-type tomato leaflets. We use the development of this method as an illustrative example and provide a detailed discussion of critical parameters and strategies that can be used to overcome technical problems that may be encountered when using this approach to purify other native proteins with different properties from plants.

## 2 Materials, equipment, and methods

### 2.1 Plant materials and growth conditions

Tomato (*S. lycopersicum* L.) cv. Ailsa Craig was used for all experiments. Seeds were germinated on moistened filter paper in Petri dishes in continuous 100 μmol m^-2^ s ^-1^ white light at 24 °C for 4 d in an environmentally controlled chamber. Tomato seedlings were transplanted to perlite, vermiculite, peat, and coconut chaff (Shandong Shangdao Biotechnology Co., Ltd., China); sphagnum peat (Pindstrup, Ryomgaard Denmark); and vermiculite mixed in a ratio of 1:1:1 and grown in an environmentally controlled room at 24 °C in a photoperiod containing 16 h of 100 μmol m^-2^ s^-1^ white light and 8 h of dark.

### 2.2 Expression and purification of SlREC2 Δ1-1463

A fragment of a cDNA clone that encodes residues G1464 to S1861 of SlREC2 (UniProt accession number A0A3Q7H2Y1; **Supplementary Figure 1**) was cloned into pHIS8-3, a derivative of pET-28a(+) with the typical hexahistidyl tag (QIAexpressionist, 2003) extended to an octahistidyl tag (Jez et al., 2000), and was expressed as a His-tagged protein in *E. coli* strain BL21-Codon Plus (DE3)-RIPL (Stratagene, Santa Clara CA). Bacteria were grown in Terrific Broth (12 g tryptone, 24 g yeast extract, and 4 mL of glycerol per liter buffered with 170 mM KH_2_PO_4_ and 720 mM K_2_HPO_4_). Expression of a His-tagged fragment of SlREC2 (His-tagged SlREC2 Δ1-1463) was induced for 4 h by adding 1 mM isopropyl β-D-1-thiogalactopyranoside (IPTG) to each culture when the OD_600_ = 0.8 to 1.0. All the following steps were performed at 4 °C. Bacteria were harvested by centrifugation at 5,000 × *g*. Approximately 23 g of bacteria were obtained from 4 L of bacterial culture and resuspended in 10 to 20 mL of buffer A (50 mM Tris-HCl, pH 8.0, 300 mM NaCl, 2 mM MgCl_2_, 20 mM imidazole, 20 mM β-mercaptoethanol, and 20% glycerol) per gram of bacterial pellet. Benzonase (Mei5bio, Beijing China) was added to a final concentration of 3U/mL with slow stirring to digest the DNA that is released when the bacteria are lysed and, therefore, to reduce the viscosity of the lysate. After a few minutes of stirring, hen egg white lysozyme (Sigma-Aldrich, St. Louis MO) dissolved in buffer A was added to a final concentration of 1 mg/mL with slow stirring. The mixture was slowly stirred for 30 min to allow for the digestion of the cell walls. Triton X-100 was slowly stirred into the mixture to a final concentration of 1% to solubilize any remaining protoplasts. This treatment with lysozyme, benzonase, and Triton X-100 converted the bacterial suspension from a turbid suspension into a transparent and particle-free solution with a consistency that appeared similar to buffer A. The treated lysate was centrifuged at 20,000 × *g* for 30 min. This method for lysing *E. coli* does not require expensive equipment, such as an ultrasonic homogenizer or a French press.

The His-tagged protein in the clarified supernatant was batch-bound to Ni-NTA agarose (Qiagen, Hilden Germany) that was equilibrated in buffer B (20 mM Tris-HCl, pH 8.0, 500 mM NaCl, 20 mM imidazole, 20 mM β-mercaptoethanol, and 20% glycerol) containing 1% Triton X-100 by mixing the supernatant and an appropriate amount of Ni-NTA agarose (i.e., a quantity that binds most of the His-tagged protein) on a tube mixer for 1 h. Ni-NTA agarose typically binds 5 to 10 mg of His-tagged protein per mL, but binding capacity is protein dependent. His-tagged proteins have a higher affinity for Ni-NTA agarose than *E. coli* proteins that may bind unoccupied sites and therefore contaminate the eluted fraction. Therefore, saturating Ni-NTA agarose with His-tagged protein is a good strategy for reducing the amount of *E. coli* proteins that contaminate the eluted fraction (QIAexpressionist, 2003). The amount of Ni-NTA required to bind most of the His-tagged SlREC2 Δ1–1463 was established empirically by testing how much His-tagged SlREC2 Δ1–1463 was not bound by the Ni-NTA agarose following the batch-binding procedure. We found that 4 mL of Ni-NTA agarose (i.e., the amount of Ni-NTA agarose in 8 mL of the 50% slurry provided by Qiagen) could bind almost all the His-tagged SlREC2 Δ1–1463 produced by a 2 L bacterial culture. The Ni-NTA agarose was batch-washed three times with buffer B containing 1.0% Triton X-100 and then batch-washed three times with buffer B using a tube mixer and centrifugation at 1,000 × *g* for 5 min in a table-top centrifuge. The washed Ni-NTA-agarose was poured into an Econo-Pac column (Bio-Rad, Hercules CA). Immediately after all the buffer B entered the column, the His-tagged SlREC2 Δ1–1463 was step eluted using buffer C (20 mM Tris-HCl, pH 8.0, 500 mM NaCl, 500 mM imidazole, 20 mM β-mercaptoethanol, and 20% glycerol). We used absorbance at 280 nm and an extinction coefficient calculated based on the amino acid sequence of His-tagged SlREC2 Δ1–1463 using ProtParam (Gasteiger et al., 2005) to estimate a yield of 15.9 mg. The His-tagged SlREC2 fragment was dialyzed repeatedly against buffer D (20 mM Tris-HCl, pH 7.9, 100 mM NaCl, 1 mM EDTA, 1 mM DTT, 20% glycerol) and then applied to a HiPrep Q HP 16/10 column (Cytiva, Marlborough USA) that was equilibrated in buffer D at a flow rate of 1 mL/min. Glycerol and low temperatures increase buffer viscosity. Therefore, using glycerol and low temperatures, such as the 4 °C used here, necessitates reduced flow rates to avoid exceeding column backpressure limits. The His-tagged SlREC2 fragment was eluted from the column with a 20-column-volume linear gradient to 100% buffer E (20 mM Tris-HCl, pH 7.9, 500 mM NaCl, 1 mM EDTA, 1 mM DTT, 20% glycerol) at a flow rate of 1 mL/min using an ÄKTA pure chromatography system (Cytiva). One-mL fractions were pooled if they contained SlREC2 Δ1-1463, dialyzed extensively against phosphate-buffered saline, concentrated with an Amicon Ultra ultrafiltration unit with a molecular weight cutoff of 10 kDa (Millipore Corporation, Billerica MA, catalog number UFC 901096), and used to develop polyclonal antisera in New Zealand white rabbits at Frdbio (Wuhan, China).

### 2.3 Affinity purification of anti-SlREC2 Δ1–1463 antibodies and immunodetection of SlREC2

The affinity purification of the anti-SlREC2 Δ1–1463 antibodies was performed at 4 °C essentially as described previously (Larkin et al., 2016). Briefly, total IgGs were purified from the antisera using Affi-Gel protein A (Bio-Rad, Hercules California) as described previously (Harlow and Lane, 1999). Next, anti-SlREC2 Δ1–1463 antibodies were affinity purified from the total IgG fraction using a resin that was constructed by linking the purified SlREC2 Δ1–1463 fragment to NHS-activated Sepharose 4 Fast Flow as recommended by the manufacturer (Smart-Lifesciences, Changzhou China). Similar resins can be constructed by linking antigens to Affi-Gel 10 or 15 (Bio-Rad) as described previously (Larkin and Guilfoyle, 1998; Larkin et al., 2003; Adhikari et al., 2009; Larkin et al., 2016). The procedure for washing and eluting the anti-SlREC2 Δ1–1463 antibodies from the SlREC2 Δ1–1463 Sepharose column was the same as described below for the purification of native SlREC2 using immunoaffinity chromatography. Immunoblotting of SlREC2 was performed essentially as described for REC1 (Larkin et al., 2016). Alternatively, fractions were sometimes analyzed more rapidly by dot blotting. Dot blots were created by spotting 5 μL of each fraction onto a nitrocellulose 0.45 mm HATF membrane (Merck Millipore, Burlington MA) and allowing the liquid to evaporate prior to immunodetection. Anti-actin antibodies were from Proteintech Group Inc. (Rosemont, IL). The amounts of protein detected in bands on immunoblots were quantified using ImageJ (Schneider et al., 2012), which quantifies the intensity of pixels in a band from a digital image of an immunoblot. The pixels are quantified using a grayscale scale. Therefore, the values produced by this process are called gray values.

### 2.4 Preparation and analysis of whole-organ extracts using denaturing conditions

Proteins were extracted using denaturing conditions as described previously (Larkin et al., 2016). Briefly, plant organs that were flash frozen using liquid nitrogen were powdered without thawing using a Tissurelyser-48 (Shanghai Jingxin Industrial Development Co., Ltd., China). The frozen powder was rapidly suspended in SDS-PAGE loading buffer. The suspension was immediately heated at 100 °C for 5 min, cooled to room temperature, and clarified by centrifugation. The protein concentrations of the clarified supernatants were determined using the A660 nm assay reagent with the ionic detergent compatibility reagent (Pierce Biotechnology, Waltham Massachusetts).

### 2.5 Analytical-scale chromatographic analysis of SlREC2

The following steps were performed at 4 °C. To test whether SlREC2 might stably associate with other proteins in plant cells, 3 g of pericarp tissue from tomato fruit collected 2 d after the breaker stage was cut into small pieces, suspended in 15 mL of homogenization buffer (50 mM HEPES-NaOH, pH 7.0, 150 mM NaCl, 5 mM EDTA, 0.1% TritonX-100, 0.2% NP-40, 5 mM DTT, 1 mM PMSF, 2 mM Pefabloc SC (Roche, Basel Switzerland), one tablet of cOmplete EDTA-free protease inhibitor cocktail (Roche) per 50 mL), and homogenized using an Omni Tissue Homogenizer (Omni International Inc., Kennesaw Georgia) three times at the maximum speed for 6 to 10 s each time. For leaflets, 4 g of tomato leaflets that included the terminal leaflet, the two to four lateral leaflets closest to the terminal leaflet, and the intercalary leaflets closest to the terminal leaflet from 40-d-old plants were suspended in 20 mL of homogenization buffer and homogenized as described for pericarp tissue. The homogenates were filtered through one layer of Miracloth and clarified by centrifugation at 13,500 × *g* for 8 min. The supernatants (S13.5) were filtered with a 0.22 μm PVDF membrane syringe filter (BioFil, Indore India), and 0.5 mL was fractionated on a Superdex 200 Increase 10/300 GL column (Cytiva) that was equilibrated in phosphate-buffered saline (PBS) (2 mM KH_2_PO_4_, 10 mM Na_2_HPO_4_, pH 7.4, 137 mM NaCl, 2.7 mM KCl) at a flow rate of 0.75 mL/min. We collected 0.5 mL fractions. The void (*V*_0_) and total volumes (*V*_t_) were determined using blue dextran and vitamin B_12_, respectively. Molecular weight standards were thyroglobulin (669 kDa), ferritin (440 kDa), aldolase (158 kDa), conalbumin (75 kDa), and ovalbumin (44 kDa) (GE Healthcare, Chicago IL). The molecular weight of native SlREC2 was estimated using the partition coefficient (*K*_av_) for SlREC2 and a standard curve prepared by graphing the *K*_av_ of each standard protein as a function of the log of the molecular weight of that standard protein. *K*_av_=(*V*_e_-*V*_0_)/(*V*_t_-*V*_0_) where *V*_e_ is the volume required to elute a protein that is partially excluded by the column, as described previously (Stellwagen, 2009).

To test whether SlREC2 could be partially purified and separated from proteases using ion exchange chromatography, we suspended 4 g of tomato leaflets that were collected from 40-d-old tomato plants as described above in 20 mL of homogenization buffer and homogenized the leaflets as described above for pericarp tissue. We added a quantity of SP Sepharose Fast Flow (Cytiva) that we expected would bind most of the protein in the homogenate, based on the dynamic binding capacity of approximately 70 mg/mL for ribonuclease A indicated by the manufacturer. To test whether SlREC2 could bind and elute from SP Sepharose, a 13-mL supernatant (S13.5) was mixed with 1 mL of SP Sepharose Fast Flow that was equilibrated in homogenization buffer on a tube mixer for 7 min. The mixture was slowly poured down the side of an Econo-Pac chromatography column (Bio-Rad, USA). The column was packed and washed using gravity flow with buffer F (50 mM HEPES-NaOH, pH 7.0, 150 mM NaCl, 5 mM EDTA, 1 mM DTT, 1 mM PMSF, 2 mM Pefabloc SC, one tablet of cOmplete EDTA-free protease inhibitor cocktail per 50 mL) until protein was undetectable in the column effluent. Protein was detected using a visual Bradford assay, which was performed by mixing column effluent (in this instance 20 μL of column effluent) with 200 μL of Bradford dye reagent (Beyotime Biotech Inc., Shanghai China) to test whether the effluent could change the color of the Bradford dye solution from brown to blue. To elute SlREC2 from the SP Sepharose column, 200 μL of buffer G (50 mM HEPES-NaOH, pH 7.0, 300 mM NaCl, 5 mM EDTA, 1mM DTT, 1 mM PMSF, and 2 mM Pefabloc, one tablet of cOmplete EDTA-free protease inhibitor cocktail per 50 mL) was manually and slowly applied to the gel bed and allowed to enter the column. Immediately after buffer G dipped just below the top of the column, this procedure was repeated. Subsequently, larger volumes of buffer G were applied to the gel bed. We manually collected 0.5 mL fractions using gravity flow.

Although using HEPES with Q Sepharose conflicts with the conventional wisdom on acceptable methodology for ion exchange chromatography (Jungbauer and Hahn, 2009), we had difficulty eluting SlREC2 from Q Sepharose using a Tris-based chromatography buffer, but we readily eluted SlREC2 from Q Sepharose using a HEPES-based chromatography buffer. The experiments with Tris-based buffers were the same as described above for testing whether SlREC2 bound SP Sepharose using 4 g of tomato leaflets and 20 mL of homogenization buffer except that in one experiment, homogenization buffer contained 50 mM Tris-HCl, pH 7.5, instead of HEPES-KOH, pH 7.0, and in another experiment, homogenization buffer contained 100 mM Tris-HCl, pH 8.0, and 100 mM NaCl instead of HEPES-KOH, pH 7.0, and 150 mM NaCl. We rapidly diluted the eluate from the SP Sepharose column with an equal volume of buffer H (50 mM HEPES-NaOH, pH 7.0, 5 mM EDTA, 1 mM DTT, 1 mM PMSF, 2 mM Pefabloc, and one tablet of cOmplete EDTA-free protease inhibitor cocktail per 50 mL). Although we did not measure the conductivity of the diluted fraction, in particular situations (e.g., when the POI elutes at a salt concentration slightly higher than the binding buffer) a conductivity meter can be used to test whether the conductivity of the diluted fraction is equivalent to the conductivity of the binding buffer. The diluted fraction was mixed with 200 μL of Q Sepharose Fast Flow (Cytiva) that was equilibrated in buffer F on a tube mixer for 7 min. We expected that this quantity of Q Sepharose Fast Flow (Cytiva) would bind most of the protein in the homogenate, based on the dynamic binding capacity of approximately 42 mg/mL for BSA indicated by the manufacturer. We slowly poured the mixture down the side of a Poly-Prep chromatography column (Bio-Rad, USA). Buffer F and gravity flow were used to pack and wash the column until no detectable protein eluted using the visual Bradford assay described above. The column was eluted by slowly adding 100 μL of buffer G to the gel bed and allowing all buffer G to enter the gel bed. This procedure was repeated three times and was followed by applying a large amount of buffer G to the Q Sepharose column. We manually collected 0.2 mL fractions from the column using gravity flow.

### 2.6 Purification of SlREC2 from tomato leaflets using immunoaffinity chromatography

The following steps were performed at 4 °C. One hundred and twenty g of tomato leaflets were collected from 38-d-old tomato plants as described above and were suspended in 500 mL of homogenization buffer in a blender and homogenized at 30,000 r/min (the highest speed) three times for 10–15 s each time. The homogenate was filtered using one layer of Miracloth and clarified at 13,500 × *g* for 8 min. The supernatant was mixed with 35 mL of SP Sepharose Fast Flow that was equilibrated in homogenization buffer on a tube mixer for 6 min and then slowly poured down the side of a 50 mm × 178 mm column (Biocomma, Shenzhen China). Homogenization buffer drained from this short and wide column relatively quickly and was washed with buffer F using gravity flow until no protein eluted from the column, as determined using the visual Bradford assay described above. Flow rates can be controlled and increased using a siphon with a reservoir containing a wash buffer (e.g., buffer F) located above the column. SlREC2 was eluted by slowly applying 10 mL of buffer G to the top of the gel bed without disturbing the gel bed and allowing buffer G and eluted proteins to flow into the column. This procedure was repeated after buffer G dipped just below the top of the column. Subsequently, a large volume of buffer G was applied to the gel bed. Fractions of 20 mL, 40 mL, 40 mL, 30 mL, and 30 mL were collected using gravity flow and tested for the presence of protein using the visual Bradford assay described above. Usually, the two 40 mL fractions contained protein and were pooled.

The protein-containing eluate from the SP Sepharose column was diluted by slowly stirring an equal volume of buffer H into the eluate from the SP Sepharose column. The diluted fraction was mixed with 7 mL of Q Sepharose Fast Flow (Cytiva) that was equilibrated in buffer F on a tube mixer for 6 min and then poured into a 25 mm × 138 mm column (Biocomma). The Q-Sepharose was washed with buffer F until no detectable protein eluted from the column, as described for SP Sepharose. Protein was eluted from Q-Sepharose by slowly adding 1 mL of buffer G to the top of the gel bed without disturbing the gel bed and allowing buffer G to flow into the gel. This procedure was repeated after buffer G dipped just below the top of the column. Subsequently, a large volume of buffer G was applied to the gel bed, and 2 mL fractions were collected using gravity flow. Protein-containing fractions were detected using the visual Bradford assay described above.

The eluate from the Q Sepharose column was typically 24 mL and was concentrated to 5 mL using an Amicon Ultra ultrafiltration unit with a 30-kDa molecular weight cutoff (Millipore). The concentrated eluate was fractionated on a HiPrep 26/60 Sephacryl S-300 High Resolution gel filtration column (Cytiva) that was equilibrated in PBS at a flow rate of 1.0 mL/min using an ÄKTA pure chromatography system (Cytiva). We collected 4-mL fractions. The void and internal volumes were determined as described above for the Superdex 200 Increase 10/300 GL column. The molecular weight standards were thyroglobulin (669 kDa), ferritin (440 kDa), aldolase (158 kDa), and conalbumin (75 kDa). The molecular weight of native SlREC2 was estimated using a partition coefficient (*K*_av_) as described above and previously (Stellwagen, 2009).

An anti-SlREC2 affinity column was constructed by covalently linking approximately 0.44 mg of affinity-purified anti-SlREC2 Δ1–1463 antibodies to rProtein A Beads 4FF (Smart-Lifesciences, Changzhou China) using dimethyl pimelimidate (Thermo Fisher Scientific, Waltham MA) as described previously (Harlow and Lane, 1999). The immunoaffinity resin was poured into a Poly-Prep column (Bio-Rad). The immunoaffinity column had a bed volume of 0.2 mL and was prewashed with 20 column volumes of 10 mM Tris-HCl, pH 7.5; 10 volumes of 100 mM glycine, pH 2.5, 50% ethylene glycol; and at least 20 column volumes of PBS. After washing with PBS, the pH of the effluent was determined to be neutral using pH paper. Typically, 7 fractions from the gel filtration column were pooled and applied to the prewashed immunoaffinity column at a flow rate of approximately 11 s per drop. This approximately four-hour process was repeated twice. The column was washed using PBS until no protein eluted, as determined using the visual Bradford assay described above, and then with 20 column volumes of 10 mM Tris-HCl, pH 7.5. SlREC2 was eluted from the column by applying 200 μL of 100 mM glycine, pH 2.5, 50% ethylene glycol to the gel bed. Immediately after all the 100 mM glycine, pH 2.5, 50% ethylene glycol entered the column, another 200 μL of 100mM glycine, pH 2.5, 50% ethylene glycol was applied. Immediately after the second aliquot of 100 mM glycine, pH 2.5, 50% ethylene glycol entered the column, 2 mL of 100 mM glycine, pH 2.5, 50% ethylene glycol was slowly applied to the column without disturbing the gel bed. Three-drop fractions (approximately 120 μL) were collected manually in 0.5 mL microfuge tubes containing 13 μL of 1 M Tris-HCl, pH 8.0, to neutralize the eluted fractions. After all protein was eluted (typically 11 fractions), the column was immediately washed with PBS until the pH of the effluent was determined to be neutral using pH paper. Importantly, this same procedure for prewashing and eluting was used to affinity purify anti-SlREC2 Δ1–1463 antibodies. Therefore, native SlREC2 should quantitatively elute from these immunoaffinity columns. The eluates from the immunoaffinity columns were analyzed using SDS-PAGE, stained with Coomassie blue. Total protein was quantified in whole-leaflet extracts prepared using non-denaturing conditions and in column fractions using the Bradford assay with BSA as a standard protein (Bradford, 1976).

Proteins were identified from Coomassie blue-stained gels using mass spectrometry at the Technology Center for Protein Sciences at Tsinghua University (Beijing, China). Briefly, gel bands, parts of lanes, or entire lanes were excised from gels for in-gel digestion. Disulfide bonds were reduced with 25 mM dithiothreitol (DTT) and alkylated with 55 mM iodoacetamide. In-gel digestion was performed using sequencing grade-modified trypsin in 50 mM ammonium bicarbonate at 37 °C overnight. The peptides were extracted twice using an aqueous solution containing 1% trifluoroacetic acid in 50% acetonitrile for 30 min. The peptide extracts were then centrifuged in a SpeedVac to reduce the volume.

For LC-MS/MS analysis, peptides were separated using a 120 min gradient elution at a flow rate of 0.300 μL/min with a Thermo-Dionex Ultimate 3000 HPLC system, which was directly interfaced with a Thermo Orbitrap Fusion mass spectrometer. The analytical column was a homemade fused silica capillary column (75 μm ID, 150 mm length; Upchurch, Oak Harbor, WA) packed with C-18 resin (300 A, 5 μm; Varian, Lexington, MA). Mobile phase A consisted of 0.1% formic acid. Mobile phase B consisted of 100% acetonitrile and 0.1% formic acid. The Orbitrap Fusion mass spectrometer was operated in the data-dependent acquisition mode using the Xcalibur 3.0 software. There was a single full-scan mass spectrum in the Orbitrap (350–1550 m/z, 120,000 resolution) followed by 3 seconds of data-dependent MS/MS scans in an Ion Routing Multipole at 30% normalized collision energy (HCD). The MS/MS spectra from each LC-MS/MS run were searched against the selected database using the Proteome Discovery searching algorithm (version 1.4).

### 2.7 Purification of SlREC2 from tomato leaflets using immunoprecipitation

Ten g of tomato leaflets were collected from 35-d-old tomato plants as described above and were suspended in 50 mL of homogenization buffer in a CD-BL01 juicer (Qcooker, China). The leaflets were homogenized three times for 8 s at 24,000 r/min each time. The homogenate was filtered through one layer of Miracloth and clarified at 30,000 × *g* for 5 min at 4 °C. SlREC2 was partially purified from the clarified supernatant using SP Sepharose and Q Sepharose as described above, except that SlREC2 was eluted from Q Sepharose using buffer G without DTT. One mL of the partially purified SlREC2 was mixed with 1.64 μg of affinity-purified anti-SlREC2 antibodies, incubated on ice for 2 h, and then mixed on a tube mixer with 20 mL of rProtein A Beads 4FF that was equilibrated in Buffer G lacking DTT for 1 h. The beads were collected using centrifugation at 2,000 × *g* for 5 min and washed 6 times by suspending the beads in buffer G lacking DTT and collecting the beads using centrifugation at 2,000 × *g* for 5 min. After aspirating the supernatant, the beads were suspended in 25 μL of 2×SDS-PAGE loading buffer, mixed well, heated at 100 °C for 5 min, and cooled to room temperature. The beads were pelleted using centrifugation. The supernatants were analyzed using SDS-PAGE and mass spectrometry as described above for the eluates from the immunoaffinity columns.

### 2.8 Other resins evaluated for SlREC2 purifications

To test whether other resins were useful for purifying SlREC2, we first separated SlREC2 from proteases and partially purified SlREC2 on SP Sepharose using 4 g of tomato leaflets and 20 mL of homogenization buffer as described above. We rapidly diluted the eluate from the SP Sepharose column (i.e., the SP 300) with an equal volume of buffer H. The diluted fraction was mixed with 200 μL of Heparin Sepharose 6 Fast Flow (Cytiva) that was equilibrated in buffer F on a tube mixer for 7 min. We slowly poured the mixture down the side of a Poly-Prep chromatography column (Bio-Rad). Buffer F and gravity flow were used to pack and wash the column. The column was eluted first using buffer G and subsequently using buffer I (50 mM HEPES-NaOH, pH 7.0, 600 mM NaCl, 5 mM EDTA, 1 mM DTT, 1 mM PMSF, and 2 mM Pefabloc, one tablet of cOmplete EDTA-free protease inhibitor cocktail per 50 mL).

We tested whether SlREC2 might bind blue Sepharose by diluting an SP 300 fraction with buffer H essentially as described for the experiments with heparin Sepharose except that the final NaCl concentration in the diluted fraction was 100 mM. The diluted SP300 was mixed with 200 μL of blue Sepharose obtained from a 1 mL HiTrap Blue HP column (Cytiva) that was equilibrated in buffer J (50 mM HEPES NaOH, pH 7.0, 100 mM NaCl, 5 mM EDTA, 1 mM DTT, 1 mM PMSF, 2 mM Pefabloc, and one tablet of cOmplete EDTA-free protease inhibitor cocktail per 50 mL) on a tube mixer for 7 min. We slowly poured the mixture down the side of a Poly-Prep chromatography column (Bio-Rad, USA). Buffer J and gravity flow were used to pack and wash the column. The column was eluted using buffer G, subsequently with buffer I and finally with buffer K (50 mM HEPES-NaOH, pH 7.0, 1 M NaCl, 5 mM EDTA, 1 mM DTT, 1 mM PMSF, and 2 mM Pefabloc, one tablet of cOmplete EDTA-free protease inhibitor cocktail per 50 mL).

We tested whether SlREC2 might bind HiTrap Capto MMC and HiTrap Capto adhere columns (Cytiva) by first purifying SlREC2 using SP Sepharose and Q Sepharose as described above in our procedure for purifying SlREC2 from tomato leaflets using immunoaffinity chromatography. The 20-mL Q Sepharose eluate was filtered and loaded onto a 1-mL HiTrap Capto MMC column that was equilibrated in buffer L (50 mM HEPES-NaOH, pH 7.0, 300 mM NaCl, 5 mM EDTA, 1 mM DTT) at a flow rate of 1.0 mL/min using an ÄKTA pure chromatography system (Cytiva). SlREC2 was eluted with 40-column volume linear gradient to buffer M (50 mM HEPES-NaOH, pH 7.0, 300 mM NaCl, 0.5 M arginine, 5 mM EDTA, 1 mM DTT). We pooled eleven 1-mL fractions containing SlREC2 and concentrated the pooled fractions to 5 mL using an Amicon Ultra ultrafiltration unit with a 30-kDa molecular weight cutoff (Millipore). The concentrated eluate was fractionated on a HiPrep 26/60 Sephacryl S-300 High Resolution gel filtration column that was equilibrated in buffer N (50 mM Tris-HCl, pH 7.5, 150 mM NaCl, 5 mM EDTA, 1 mM DTT) at a flow rate of 1.0 mL/min using an ÄKTA pure chromatography system (Cytiva). We collected eight 4-mL fractions containing SlREC2 and loaded the pooled fractions onto a 1-mL HiTrap Capto adhere column (Cytiva) that was equilibrated in buffer N at a flow rate of 1.0 mL/min using an ÄKTA pure chromatography system (Cytiva). SlREC2 was eluted with a 30-column volume linear gradient to buffer O (50 mM Tris-HCl, pH 7.5, 150 mM NaCl, 1 M arginine, 5 mM EDTA, 1 mM DTT).

## 3 Results and discussion

### 3.1 Development and affinity purification of anti-SlREC2 Δ1–1463 polyclonal antibodies

If no convenient enzyme assay is available, immunoblotting with affinity-purified anti-POI antibodies is an effective method for following POIs during purifications. Additionally, affinity-purified anti-POI antibodies are necessary for immunoaffinity chromatography and immunoprecipitation. Animals have the capacity to produce large numbers of antibodies that bind distinct antigens with sufficient affinity and are commonly used as affinity reagents for the purification of POIs. There are numerous commercial resources for producing and using antibodies for this purpose (Harlow and Lane, 1999). Although numerous methods are available to produce a POI for antibody production, expressing recombinant proteins in prokaryotic expression systems is relatively simple and economical (PouresmaeilAzizi-Dargahlou, 2023). We prefer to develop antibodies against proteins that were expressed in *E. coli* as His-tagged proteins because plasmids and *E. coli* strains are available that express extremely high levels of His-tagged proteins, His-tagged proteins can be purified using both non-denaturing and denaturing conditions, and His tags are only very weakly immunogenic (Loughran et al., 2023). For the development of polyclonal antisera, the recombinant POI should be highly purified to avoid developing antibodies against contaminants. If the POI is fused to an immunogenic tag (e.g., a large protein, such as glutathione *S*-transferase or maltose-binding protein), the tag should be removed prior to immunization to avoid developing antibodies primarily against the tag. A variety of strategies can be employed to optimize the expression of soluble proteins in *E. coli*, including altering the temperature during production; using different strains, tags, and promoters; and expressing proteins in the cytoplasm, periplasm, or secretion into the growth medium (PouresmaeilAzizi-Dargahlou, 2023). Coexpressing a POI with molecular chaperones may also facilitate the production of properly folded proteins (Fatima et al., 2021), although particular molecular chaperones may be required for the proper folding of some proteins (Aigner et al., 2017). Expressing a partial POI containing a small number of predicted domains or only one predicted domain in *E. coli* may facilitate the production of a recombinant protein suitable for antibody development if expressing a full-length recombinant protein in *E. coli* is not possible. If expressing even a single domain in *E. coli* is impossible, an alternative is to develop antisera against peptides or small proteins synthesized *in vitro* (Trier et al., 2019).

Affinity-purified polyclonal antibodies are useful for immunoaffinity purifications because they may bind numerous epitopes on the POI, and therefore, the protein-protein interactions that maintain a multisubunit protein complex will probably not obscure all epitopes recognized by polyclonal antibodies. Although monoclonal antibodies are also useful for immunoaffinity purifications, the time and cost of developing monoclonal antibodies are considerably higher relative to polyclonal antisera. However, because of the complexity of the interactions between polyclonal antibodies and their antigens, denaturing conditions are required to separate antigens from polyclonal antibodies. In contrast, in addition to requiring smaller amounts of a less pure antigen, mild conditions that may maintain enzyme activity and the integrity of multisubunit protein complexes may be used to disrupt the interactions between antigens and particular monoclonal antibodies because monoclonal antibodies bind single epitopes (Burgess and Thompson, 2002). For polyclonal antibody development, immunization with intradermal injections can induce a more robust immune response than either subcutaneous or intramuscular injections (Schnyder et al., 2021) and therefore should be considered. If raising antibodies is impossible (e.g., if the antigen is toxic to animals or not immunogenic), affinity resins suitable for purifying a POI may be developed using less commonly used alternative affinity reagents, such as RNA, DNA, or peptide aptamers (Trier et al., 2019; Chu et al., 2021); molecularly imprinted polymers (Trier et al., 2019); or various types of protein scaffolds (Chu et al., 2021; Mouratou and Pecorari, 2022).

**Figure 1:**
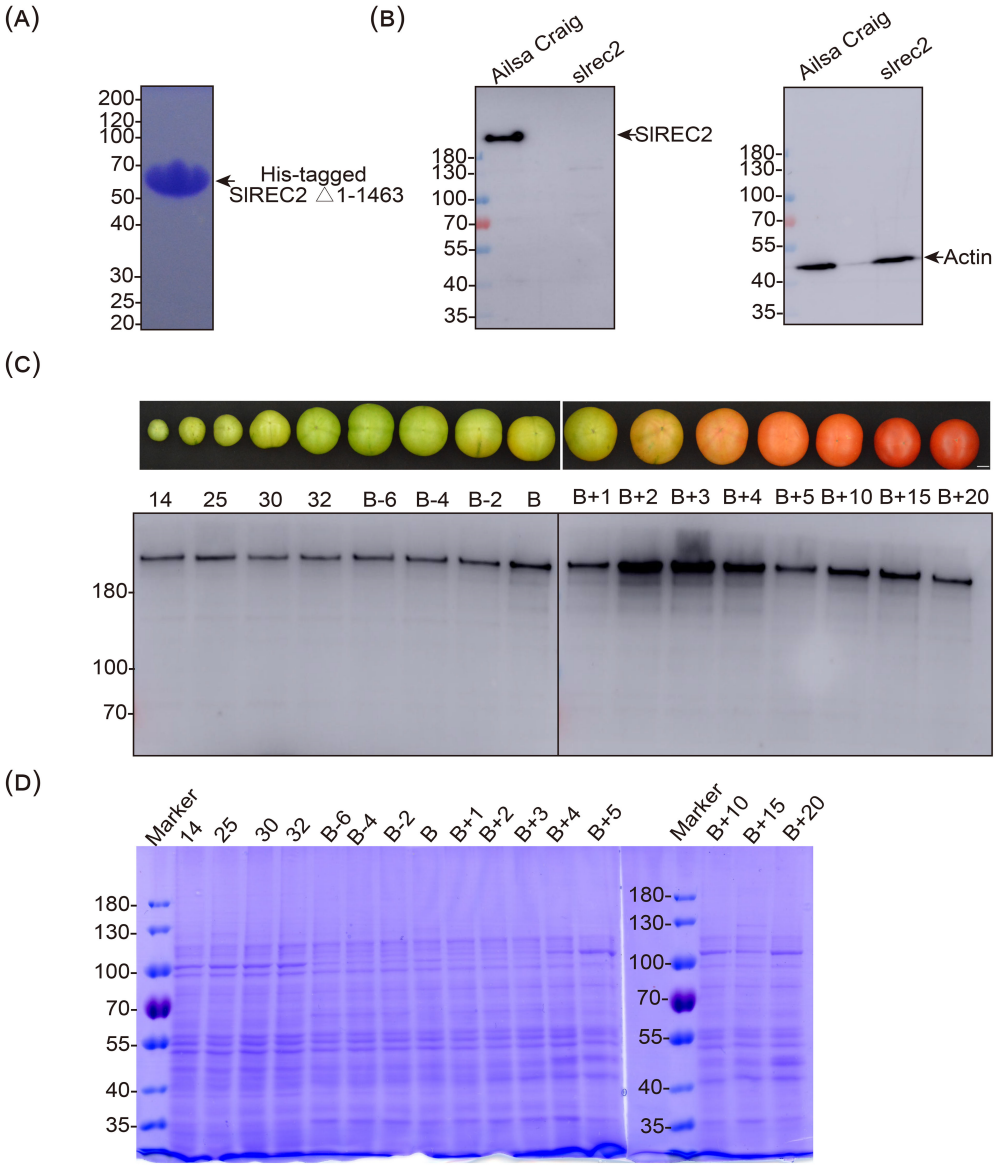
Development of affinity-purified anti-SlREC2 Δ1–1463 antibodies. **(A)** Purified SlREC2 Δ1-1463. Twenty μg of purified SlREC2 Δ1–1463 was analyzed using a 7.5% SDS gel that was stained with Coomassie brilliant blue R-250. The mass of each standard protein is indicated at the left in kDa. **(B)** Immunoblotting analysis of fruit pericarp extracts from wild type and *slrec2*. Whole-pericarp extracts were prepared from the equatorial region of fruit collected 5 d after the breaker stage. Total protein was extracted using denaturing conditions. Two μg of protein from wild type (Ailsa Craig) and *slrec2* was analyzed using a 7.5% SDS gel and immunoblotting with affinity-purified anti-SlREC2 Δ1–1463 antibodies (left). A replicate gel was analyzed using anti-actin antibodies (right). Mass standards are indicated on each blot at the left. **(C)** Expression pattern of SlREC2 during the development and ripening of tomato fruit. Total protein was extracted from the equatorial region of fruit pericarps using denaturing conditions at the indicated number of d post anthesis (i.e., 14, 25, 30, and 32) and at the indicated number of d before and after the breaker stage **(B)**. Ten μg of protein was analyzed at each stage of fruit development and ripening using a 7.5% gel and immunoblotting with affinity-purified anti-SlREC2 Δ1–1463 antibodies. **(D)** Total protein in developing and ripening tomato fruit. Replicates of the gels used to produce the immunoblot shown in **(C)** were stained with Coomassie brilliant blue.

We developed polyclonal antisera against our POI, SlREC2, which has a calculated mass of 202 kDa and is homologous to four other proteins encoded by the tomato genome (**Supplementary Figure 1**). To raise antibodies that specifically recognize SlREC2, we expressed a 44-kDa carboxy-terminal fragment of SlREC2 (i.e., G1464 to S1861) that shares only low levels of amino acid sequence similarity with the four SlREC2 homologues from tomato (Hu et al., 2024; **Supplementary Figure 1**) and purified this SlREC2 fragment. We could not detect contaminating proteins when we analyzed 20 μg of the purified His-tagged SlREC2 fragment using a Coomassie blue-stained SDS gel and therefore conclude that the purity was greater than 99% (**Figure 1A**). The affinity-purified anti-SlREC2 Δ1–1463 antibodies that we developed specifically recognized a single band that migrated in an SDS gel as expected when a tomato fruit pericarp extract prepared using denaturing conditions was analyzed using immunoblotting with affinity-purified anti-SlREC2 Δ1–1463 antibodies (**Figure 1B**). Immunoreactive bands were barely detectable in an *slrec2* null mutant (**Figure 1B**), which contains a frameshift mutation near the beginning of the coding sequence of *SlREC2*. Moreover, SlREC2 was expressed at all stages of the development and ripening of tomato fruit and accumulated to elevated levels during the early stages of ripening (**Figures 1C, D**), which is consistent with the expression pattern of the mRNA encoding SlREC2 (Hu et al., 2024). Although we readily detected SlREC2 in whole-cell extracts prepared from fruit pericarps using immunoblotting (**Figure 1C**), we could not detect a major band migrating as expected for a 200-kDa protein in replicate Coomassie blue-stained SDS gels (**Figure 1D**). We suggest that SlREC2 is not a highly abundant protein and that immunoblotting with our anti-SlREC2 antibodies is a more sensitive method for detecting SlREC2 in whole-cell extracts than staining SDS gels with Coomassie blue. These data indicate that these affinity-purified anti-SlREC2 Δ1–1463 antibodies specifically bind SlREC2 on immunoblots and therefore, provide evidence that these antibodies will be useful for immunoaffinity chromatography and immunoprecipitation.

### 3.2 Extraction and characterization of native SlREC2 from tomato leaflets

The first step toward testing whether the POI stably binds other proteins *in vivo* is to determine the expression pattern and subcellular location of the POI. This information will help researchers to choose a starting material (e.g., leaves, fruits, roots, embryos, etc.) and whether the procedure can begin with the purification of an organelle or organellar fraction, which can provide a major purification of the POI. Gene expression patterns are available from publicly accessible databases for many plant species. For example, numerous databases provide information on the expression of SlREC2 (Winter et al., 2007; Matas et al., 2011; Tomato Genome Consortium, 2012; Shinozaki et al., 2018). Databases that provide at least predictions for subcellular locations are also publicly available (Hooper et al., 2017). Additionally, live cell imaging of *Nicotiana benthamiana* cells transiently expressing a POI fused to a fluorescent protein can often provide reliable information on subcellular location (Sparkes et al., 2006). However, sometimes the data obtained using this approach appear to be influenced by experimental conditions (Hu et al., 2024). Analyzing subcellular fractions using immunoblotting with affinity-purified anti-POI antibodies will provide compelling evidence for the subcellular location of a POI.

To assess whether the POI might stably associate with other proteins in plant cells, whole-organ extracts, soluble fractions from purified organelles, or solubilized membranes can be fractionated using gel filtration chromatography (Stellwagen, 2009), density gradient centrifugation (Fernandez-Martinez et al., 2016), or particular types of native gels that provide information on molecular mass, such as blue native gels (Wittig and Schägger, 2009). If the POI fractionates, sediments, or migrates with a size larger than the mass of the POI calculated from its amino acid sequence, the data provide evidence that the POI stably associates with other proteins. If practical, comparisons between the fractionation, sedimentation, or migration of the native POI extracted from wild-type plants and the full-length recombinant POI (e.g., expressed in a microorganism, such as *E. coli*) are useful because, in many instances, proteins related to the POI-BPs will not be present in the heterologous system used to produce the recombinant protein. Moreover, the recombinant POI may accumulate at much higher levels than other proteins in the heterologous system, will not interact with proteins from the heterologous system, and therefore, will be a monomer or a homomultimer. Thus, the recombinant POI will fractionate, sediment, or migrate differently than the native POI associated with POI-BPs. If the native POI does not stably bind POI BPs, the native POI and recombinant POI will fractionate, sediment, or migrate similarly. If the recombinant POI forms a complex with a protein from a heterologous system (e.g., *E. coli*) in stoichiometric amounts, the complex would probably be a different size than the native POI associated with POI-BPs that accumulates in plants. For example, comparisons between the gel filtration column elution profiles of the recombinant GENOMES UNCOUPLED 4 (GUN4) protein expressed in *E. coli* without its chloroplast transit peptide and GUN4 in partially solubilized chloroplast membranes provided evidence that GUN4 binds other proteins that associate with chloroplast membranes in Arabidopsis (Larkin et al., 2003).

**Figure 2:**
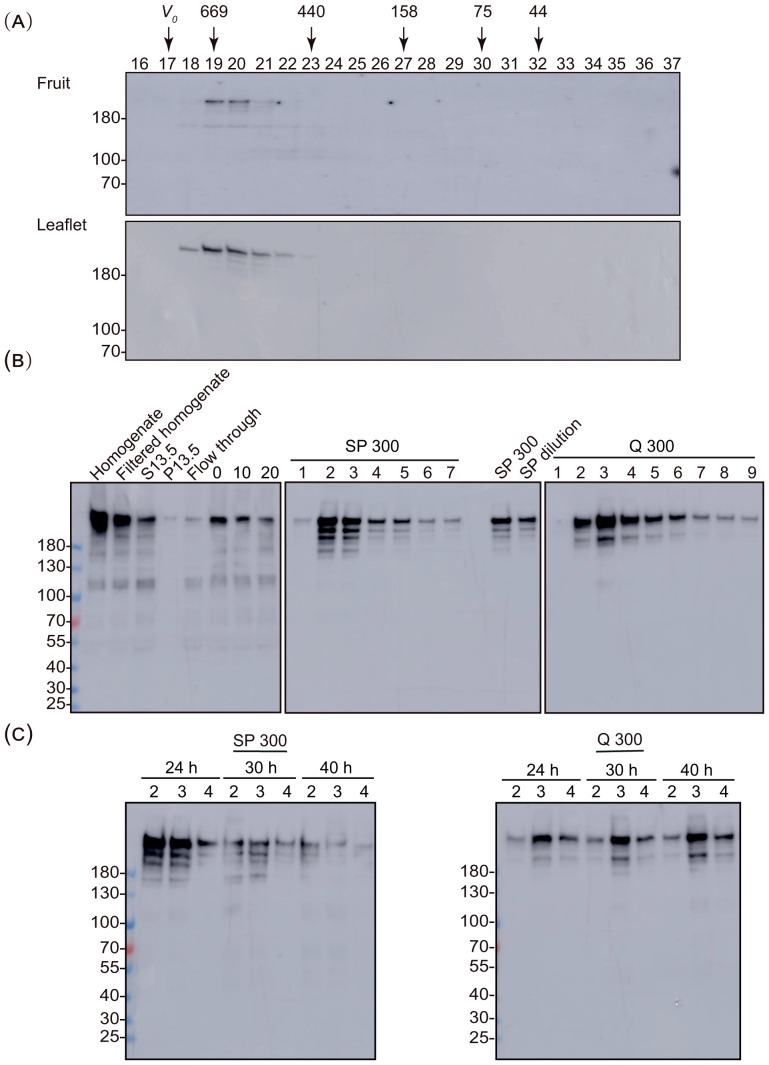
Chromatographic analysis of SlREC2. **(A)** Elution of SlREC2 from a gel filtration column. Whole-fruit pericarp extracts (top) from B+2 fruit and whole-leaflet extracts prepared from 30-d-old leaflets (bottom) were fractionated on a Superdex 200 Increase 10/300 GL column. Equal volumes of each fraction were analyzed using an 8% SDS gel and immunoblotting with affinity-purified anti-SlREC2 Δ1–1463 antibodies. The void volume (*V_0_*) is indicated. The fractions containing the elution peaks for each standard protein are indicated with the mass of the pertinent standard protein in kDa. **(B)** Binding and elution of SlREC2 from SP Sepharose and Q Sepharose. Whole-leaflet extracts prepared from 30-d-old leaflets (Homogenate) were filtered through Miracloth (Filtered homogenate) and clarified by centrifugation at 13,500 × *g* to yield supernatant (S13.5) and pellet (P13.5) fractions. Equal volumes of these fractions, the unbound (Flow through), fractions eluted from SP Sepharose and Q Sepharose using 300 mM NaCl (SP 300 and Q 300), and the SP300 diluted with buffer H (SP dilution) were analyzed using 8% SDS gels and immunoblotting with affinity-purified anti-SlREC2 Δ1–1463 antibodies. The amount of SlREC2 remaining after the S13.5 was incubated at 4 °C for 0, 10, and 20 min is shown. **(C)** Proteolysis of SlREC2 in fractions eluted from SP Sepharose and Q Sepharose. SlREC2 was purified using SP Sepharose and Q Sepharose as described in **(B)**. The amount of SlREC2 remaining after three fractions from the elution peaks (fractions 2, 3, and 4) from SP Sepharose and Q Sepharose were incubated at 4 °C for 24, 30, and 40 h is shown. Equal volumes of each fraction and at each time point were analyzed using 8% SDS gels and immunoblotting with affinity-purified anti-SlREC2 Δ1–1463 antibodies. The relative mobilities of the molecular weight standard proteins are indicated (left) in kDa.

The mRNA encoding SlREC2 is expressed in leaves and ripening fruit. An SlREC2-yellow fluorescent protein (YFP) fusion protein was reported to accumulate mostly in the cytoplasm and to a minor extent in the nucleus (Hu et al., 2024). In plant cells, most of the protein accumulates in the chloroplast and cytosol (Heinemann et al., 2021). Therefore, beginning the procedure with cytosolic fractions would not provide a large purification and would remove the nuclear isoform of SlREC2 and vice versa. Moreover, many contaminants are readily identifiable because they are at least bioinformatically predicted to reside in a distinct subcellular compartment. For these reasons, we chose to prepare whole-fruit pericarp extracts and whole-leaflet extracts from tomato to purify native SlREC2. We estimated the size of the native SlREC2 protein by fractionating these extracts on a gel filtration column. SlREC2 was detected using immunoblotting. Based on these data, we calculated the size of native SlREC2 from both fruit and leaflets was 640 kDa (**Figure 2A**; **Supplementary Figure 2**, **Supplementary Figure 3A**). Gel filtration chromatography measures the volume created by a protein as it tumbles in solution (Stellwagen, 2009). Therefore, if SlREC2 were a spherical and monomeric globular protein, SlREC2 would elute from the gel filtration column as a protein with a mass of approximately 200 kDa. Our finding that SlREC2 elutes from a gel filtration column more quickly than expected for a monomeric globular protein with a calculated mass of 202 kDa provides evidence that SlREC2 is not monomeric (i.e., stably associates with itself or with other proteins *in vivo*), is asymmetric (i.e., not spherical), or a combination of these possibilities. Because of the difficulties that are often encountered when expressing large proteins in *E. coli*, we did not compare the gel filtration column elution profiles of native SlREC2 extracted from fruits and leaflets to the full-length recombinant SlREC2 protein.

Successful extraction of a native protein requires a homogenization buffer that promotes the stability of the native protein by mimicking its cellular environment (Deutscher, 2009). Our homogenization buffer maintained a neutral pH, moderate ionic strength, and reducing environment because SlREC2-YFP accumulates in the cytoplasm and nucleus (Hu et al., 2024). When preparing a whole-cell extract to purify a protein that accumulates in the cytosol, the volume of the homogenization buffer must be sufficiently large and the concentration of the buffer must be sufficiently high to neutralize the acidity that comes from the vacuole, which can occupy most of the cellular volume (Gegenheimer, 1990). Ideally, quantitative enzyme assays would be performed, and buffers with p*K*_a_ values close to the pH optimum would be screened for buffer effects (Stoll and Blanchard, 2009). Although NaCl is commonly used to maintain ionic strength when purifying proteins, other anions and cations may more effectively promote solubility of the POI (Gregory et al., 2022). In particular, using acetate, sulfate, or glutamate rather than chloride may promote activity and stability (Deutscher, 2009; Stoll and Blanchard, 2009). Nonetheless, we found that NaCl was a suitable salt for the extraction and purification of SlREC2. A metal chelating agent, such as EDTA, may also be included to minimize heavy metal damage to proteins and to inhibit metalloproteases. We found that including low concentrations of detergents in our homogenization buffer increased our yield of soluble SlREC2 but that excluding detergents from the later steps of the procedure did not have a major impact on the yield of SlREC2. Sometimes detergents can prevent soluble proteins from sticking to surfaces during the purification procedure and therefore increase yield (Deutscher, 2009). However, including even low concentrations of detergents in homogenization buffers may increase the number of contaminating proteins in the final fraction. Additionally, detergents will influence the choice of method used to detect the elution of protein from columns because some detergents will interfere with visual dye-binding assays, such as the visual Bradford assay described above, or the detection of protein absorbance at 280 nm. Polyvinylpyrrolidone (PVP) and polyvinylpolypyrrolidone (PVPP) bind phenolics and alkaloids that may bind and inhibit the activity of proteins (Gegenheimer, 1990). However, earlier work that we conducted when trying to develop procedures for purifying REC1 from Arabidopsis (Larkin et al., 2016) provided evidence that including PVP and PVPP did not influence the purification of REC1 (LZ, unpublished data). Based on these data, we decided to not test whether PVP or PVPP might facilitate the purification of SlREC2.

### 3.3 Purification of SlREC2 from tomato leaflets

The isoelectric points of most proteins encoded by plant genomes are acidic (Mohanta et al., 2019), and therefore, at a neutral pH, fewer plant proteins will bind cation exchange resins than anion exchange resins. Therefore, using a cation exchange resin or an affinity resin is a good choice for a first step. Calculating the isoelectric point, searching for acidic and basic patches, and searching for domains or short sequence motifs with known functions can help guide the choice of ion exchange or affinity resins that the POI might bind. Although affinity resins can be constructed by covalently linking a variety of ligands to a solid support, numerous affinity resins with ligands already linked to a solid support are commercially available, such as affinity resins that utilize heparin, lectins, immobilized metals, and triazine dyes, such as Cibacron blue (Dixit, 2022; Hage et al., 2023). The influence of unknown POI-BPs on the chromatographic behavior of a POI is unpredictable, and therefore, ideally, a broad spectrum of resins should be tested. The calculated pI of SlREC2 is 5.59, which provides evidence that SlREC2 might bind anion exchange resins when the pH is neutral. However, SlREC2 may also bind cation exchangers and affinity resins due to the presence of positively charged amino acid residues in SlREC2 and the possibility of SlREC2 stably binding other proteins. Utilizing distinct procedures prior to the immunoaffinity step may lead to different populations of contaminants in the immunopurified fraction. Highly enriching a POI without the denaturing immunoaffinity purification step may be useful for testing POI activity. For these reasons, we tested whether SlREC2 might bind SP Sepharose, Q Sepharose, Heparin Sepharose, Blue Sepharose, Capto MMC, and Capto adhere (**Supplementary Figure 4**).

We found that when we extracted SlREC2 using non-denaturing conditions to preserve activity and protein-protein interactions, we detected immunoreactive bands that migrated more quickly than full-length SlREC2 (**Figures 2B, C**). These more quickly migrating bands were absent when we extracted SlREC2 using denaturing conditions (**Figures 1B, C**), presumably because these more rapidly migrating bands were produced by proteases that were inactivated when SlREC2 was extracted using denaturing conditions. The proteolytic degradation was more severe in extracts prepared from fruit than from leaves. Therefore, we choose to purify SlREC2 from leaves. SlREC2 bound both SP Sepharose and Q Sepharose in buffers containing 150 mM NaCl and eluted from both resins using step gradients with buffers containing 300 mM NaCl (**Figure 2B**; **Supplementary Figure 3B**) perhaps because SlREC2 contains both positively and negatively charged amino acid residues or perhaps because SlREC2 stably binds other proteins. We found that no additional SlREC2 eluted from either resin with buffers containing either 600 mM NaCl or 1000 mM NaCl. To protect SlREC2 from proteolytic degradation, we included protease inhibitors in the homogenization buffer and most of the chromatography buffers. Also, we found that rapid batch binding, washing, and eluting SlREC2 from SP Sepharose and then purifying SlREC2 from the eluted fractions (i.e., SlREC2 in the SP 300) on Q Sepharose not only partially purified SlREC2 but also rapidly separated SlREC2 from protease activity (**Figures 2B, C**; **Supplementary Figures 3B, C**). Although eluting proteins from columns using linear gradients provides greater resolution than step gradients, the batch-binding of POIs in large volumes of homogenates followed by eluting POIs from columns using step gradients as described here can be performed more rapidly than loading large volumes of homogenates onto prepacked columns and eluting POIs using linear gradients. Therefore, because of the possibility of greater speed, batch-binding strategies, such as the strategy described here, may facilitate the purification of protease-sensitive proteins. Using columns with large internal diameters will also facilitate high flow rates (GE Healthcare Ion Exchange Chromatography Principles and Methods). Eluting proteins from columns using step gradients also tends to produce fractions containing higher protein concentrations than linear gradients, which promotes protein stability (Deutscher, 2009). Glycerol effectively promotes stability by preventing aggregation (Vagenende et al., 2009) and therefore, in general, is useful to include in homogenization and chromatography buffers in concentrations that range from 10% to 20%. On the other hand, glycerol reduces flow rates, and therefore, consideration should be given to excluding glycerol from buffers when speed is a critical parameter, such as when purifying highly protease-sensitive proteins. Ion exchange groups linked to magnetic beads can increase the speed of these steps but are more expensive than ion exchange groups linked to Sepharose.

**Figure 3:**
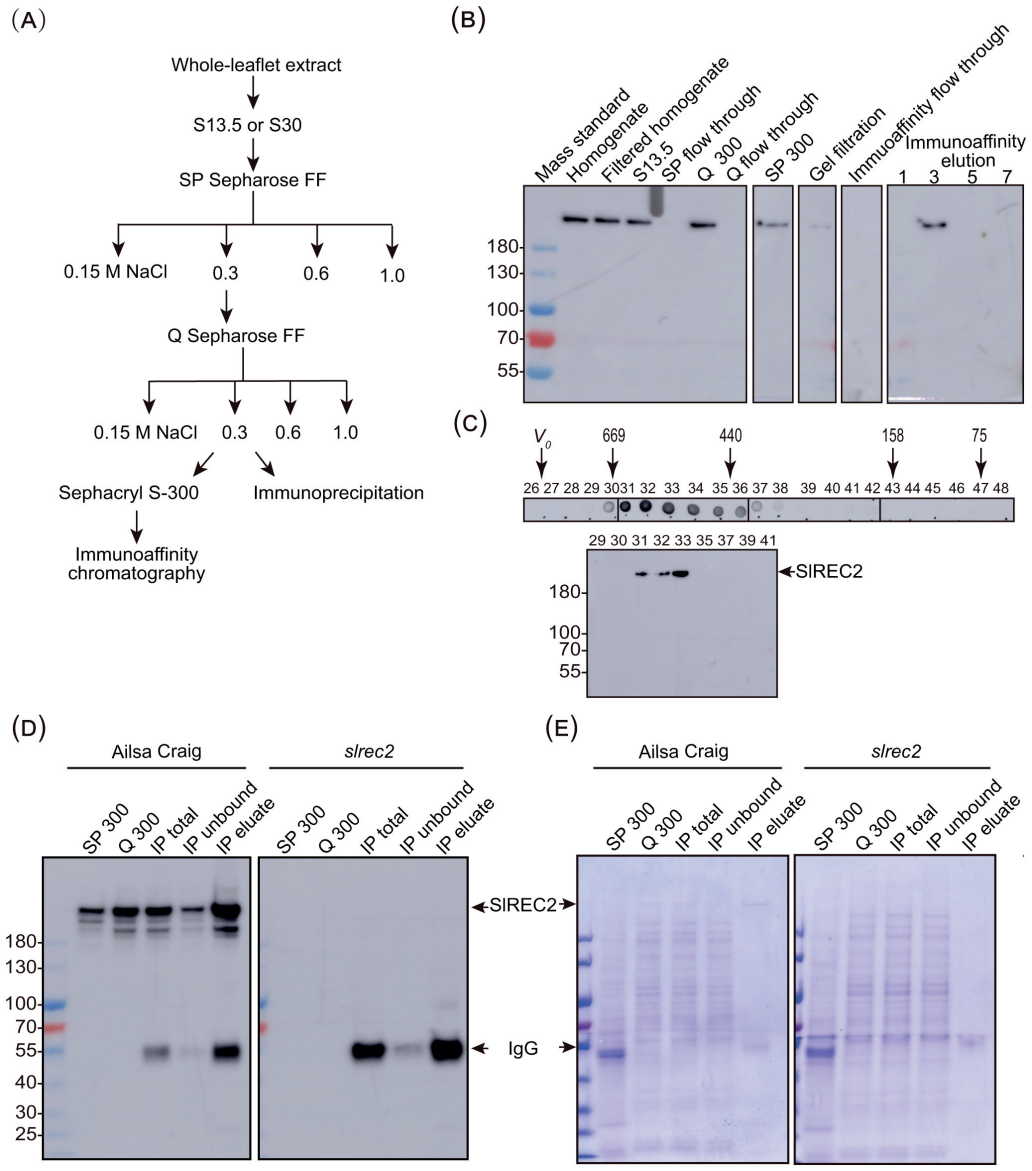
Purification of native SlREC2 from tomato leaflets. **(A)** Overall workflow for the purification of SlREC2 from leaflets. S13.5 and S30 refer to supernatants produced by centrifugation of homogenates at 13,500 × *g* and at 30,000 × *g*, respectively. **(B)** Key fractions from the purification of SlREC2 from leaflets ultimately using immunoaffinity chromatography. Whole-leaflet extracts prepared from 38-d-old leaflets (Homogenate) were filtered through Miracloth (Filtered homogenate) and clarified by centrifugation at 13,500 × *g* to yield a supernatant (S13.5). Fractions containing SlREC2 and flow-through fractions from the indicated steps are shown. SP 300 and Q 300 refer to fractions eluted from SP Sepharose and Q Sepharose, respectively, using a buffer containing 300 mM NaCl. Equal volumes of each fraction were analyzed using 6.5% SDS gels and immunoblotting with affinity-purified anti-SlREC2 Δ;1–1463 antibodies. The immunoblots were analyzed together in the same field of view for the same amount of time. **(C)** Elution of SlREC2 from a gel filtration column. The concentrated Q eluate was fractionated on a HiPrep 26/60 Sephacryl S-300 High Resolution gel filtration column. SlREC2 was detected in each eluted fraction by dot blotting 5 μL of each fraction followed by immunodetection with affinity-purified anti-SlREC2 Δ1–1463 antibodies (above). The fractions analyzed with a dot blot were spotted onto a rectangular membrane as seven rows of fractions containing six fractions per row. The boundary of each row is indicated with a black line. Equal volumes of selected fractions were analyzed using a 6.5% SDS gel and immunoblotting with affinity-purified anti-SlREC2 Δ1–1463 antibodies (below). The void volume (*V_0_*) is indicated. The fractions containing the elution peaks for each standard protein are indicated with the mass of the appropriate standard protein in kDa. The relative mobilities of molecular weight standards for the SDS gel are indicated at the left. **(D)** Key fractions from the purification of SlREC2 from leaflets ultimately using immunoprecipitation. Purifications were performed using 30-d-old leaflets from Ailsa Craig and *slrec2* as indicated. SP 300 and Q 300 were as defined in **(B)**. Affinity-purified anti-SlREC2 Δ1–1463 antibodies were added to a Q300 fraction to yield the IP total fraction. Fractions were analyzed using immunoblotting with 8% SDS gels as described in **(B)**. The relative mobilities of molecular weight standards for the SDS gel are indicated at the left. **(E)** Total protein in the key fractions from the purification of SlREC2 from leaflets ultimately using immunoprecipitation. Replicates of the gels used to produce the immunoblots shown in **(D)** were stained with Coomassie brilliant blue.

Based on these data, we developed a purification procedure that involved first fractionating whole-leaflet extracts on SP Sepharose and Q Sepharose. Because native SlREC2 is an unusually large protein, we further purified the native SlREC2 on a preparative gel filtration column prior to the immunoaffinity purification of native SlREC2. We applied the fractions that eluted from the gel filtration column to an immunoaffinity column constructed by covalently crosslinking the affinity-purified anti-SlREC2 Δ1–1463 antibodies to protein A that was linked to beads (**Figures 3A, B**; **Supplementary Figure 5**). Alternatively, we immunoprecipitated native SlREC2 from the Q Sepharose eluate using affinity-purified anti-SlREC2 Δ1–1463 antibodies (**Figures 3A, D, E**). We monitored the fractionation of native SlREC2 using immunoblotting (**Figures 3B-D**) and found that after these purifications and prior to immunoaffinity chromatography, SlREC2 eluted from a HiPrep 26/60 Sephacryl S-300 High Resolution gel filtration column as a 580-kDa protein (**Figure 3C**; **Supplementary Figure 6**), which is smaller than the size of native SlREC2 in a whole leaflet extract obtained using a Superdex 200 Increase 10/300 GL column (**Figure 2A**; **Supplementary Figure 2**). These data indicate that a native SlREC2 complex may have partially disintegrated during this purification procedure. Alternatively, the different size estimates (i.e., 640 kDa compared to 580 kDa) may be due to the use of different gel filtration columns for the different experiments (**Figures 2A**, **3C**). Total protein was not possible to detect by Coomassie blue staining of the dot blot and immunoblot shown in **Figure 3C** because the protein concentration was diluted too much by the gel filtration chromatography procedure (**Table 1**).

**Table 1:** Summary of SlREC2 purification using immunoaffinity chromatography.

Step^a^	Concentration (mg/mL)^b^	Volume (mL)	Protein (mg)	Gray value (units)^c^	Total SlREC2 (units)^d^	SlREC2 purity (units/mg)^e^	Fold Purification^f^	Yield (%)^g^
Supernatant	3.92	370	1450	1650	611000	420	1	100
SP Sepharose	0.22	90	20.2	1600	144000	7130	17	24
Q Sepharose	0.19	26	4.9	1980	51500	10500	25	8.4
Ultrafiltration	1.01	4.0	4.0	6290	25200	6300	15	4.1
Gel filtration	0.13	28	3.6	310	8700	2420	6	1.4
IAC		0.45	0.2	1420	640	3200	8	0.1

a Pertinent data is indicated for each fraction produced during the purification of SlREC2 using immunoaffinity chromatography (IAC). Values for each purification step were obtained from pooled fractions containing SlREC2.

b Protein concentrations were determined using the Bradford assay with BSA serving as the standard protein.

c The amount of SlREC2 was quantified from images of immunoblots using ImageJ as described in the materials, equipment, and methods section. Gray value refers to the intensity of the signal from a protein band in our immunoblotting assay.

d Total SlREC2 was calculated by multiplying the gray value by the volume of the pooled fractions.

e SlREC2 purity was calculated by dividing the gray value by the amount of protein in the pooled fractions.

f Fold purification was calculated by dividing SlREC2 purity for a particular pool of fractions by SlREC2 purity in the supernatant.

g Percent yield is the percent of total SlREC2 in a particular pool of fractions relative to the total SlREC2 in the supernatant.

**Figure 4:**
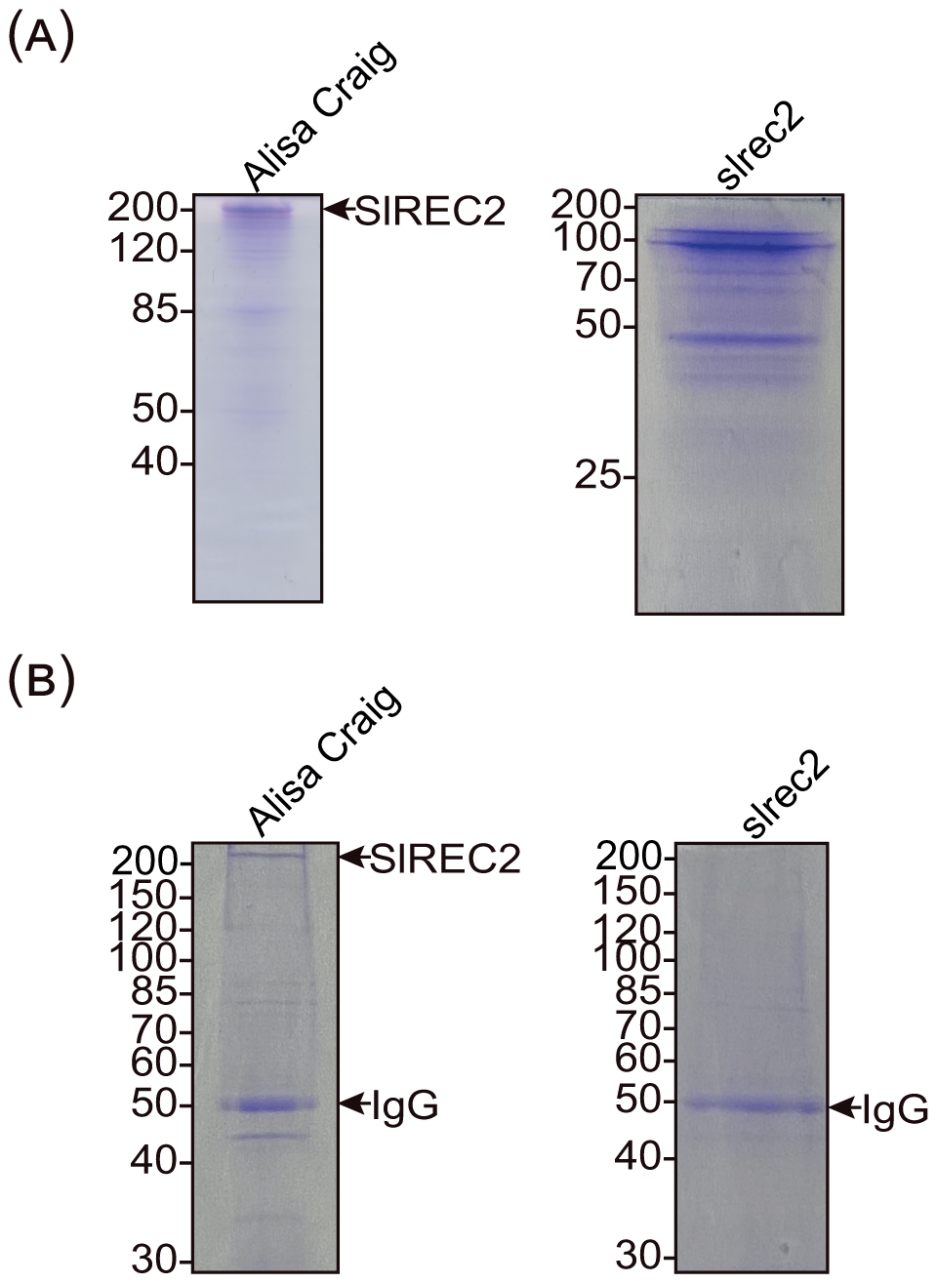
Native SlREC2 purified from tomato leaflets. **(A)** Native SlREC2 purified ultimately using immunoaffinity chromatography. The purifications were conducted using 38-d-old wild type (Ailsa Craig, left) and *slerc2* (right). Equal volumes from the total protein elution peak from the immunoaffinity columns were analyzed using 10% SDS gels that were stained with Coomassie brilliant blue. The relative mobilities of molecular weight standards are indicated at the left in kDa. **(B)** Native SlREC2 purified ultimately using immunoprecipitation. The purifications were conducted using 38-d-old leaflets from wild type (Ailsa Craig) (left) and *slerc2* (right). Protein was eluted from the immunoprecipitates using equal volumes of SDS-PAGE loading buffer followed by heating at 100 °C for 5 min. Equal volumes of eluate were analyzed using 8% SDS gels that were stained with Coomassie brilliant blue. The relative mobilities of molecular weight standards are indicated at the left in kDa.

After either type of immunoaffinity purification, we found that we purified a protein from wild-type tomato that migrated as expected in SDS gels (**Figures 4A, B**). Using mass spectrometry, we determined that this protein is SlREC2 (**Table 2**). However, we could not purify the same protein from the *slrec2* mutant (**Figures 4A, B**; **Table 2**). Based on these data, we concluded that we successfully purified SlREC2 from leaflets. The copurifying proteins that migrated more quickly than expected for full-length SlREC2 could be SlREC2-binding proteins or proteolytic degradation products of SlREC2. Indeed, a comparison of SlREC2 extracted using denaturing conditions (**Figure 1**) and non-denaturing conditions (**Figures 2**, **3**) indicates that some proteolytic degradation of SlREC2 occurred during the extraction and purification of SlREC2 using non-denaturing conditions. The copurifying proteins might also be contaminants that do not bind SlREC2. Based on these data, we cannot distinguish whether SlREC2 stably associated with other proteins or whether SlREC2 only associated with itself. The data collected using gel filtration chromatography are also subject to interpretation. Gel filtration chromatography measures the spherical volume created by a protein as it tumbles in solution (Stellwagen, 2009). Therefore, SlREC2 associating with itself or assuming an asymmetric conformation rather than the more spherical conformation of a globular protein may also contribute to SlREC2 eluting more quickly from gel filtration columns than anticipated for a monomeric protein with a calculated mass of 202 kDa. As described above, comparisons between native POIs and recombinant POIs (e.g., using gel filtration chromatography, density gradients, or blue native gels) can provide evidence that native POIs stably bind POI-BPs *in vivo*.

**Table 2:** Identification of native SlREC2 purified from wild type (Ailsa Craig) tomato leaflets using mass spectrometry.

Genotype	Method	Accession^a^	Percent sequence coverage	Unique peptides	MW (kDa)
Ailsa Craig	IAC	A0A3Q7H2Y1	60.54	147	201.6
*slrec2*	IAC	ND	ND	ND	ND
Ailsa Craig	IP	A0A3Q7H2Y1	49.92	75	201.6
*slrec2*	IP	A0A3Q7H2Y1	2.73	2	201.6

For the purification of SlREC2 ultimately using immunoaffinity chromatography (IAC), SlREC2 was identified using mass spectrometry from a gel slice containing a band that migrated in an SDS gel as expected based on the calculated mass of SlREC2 and was purified from Ailsa Craig. For *slrec2* and for purifications that ultimately used immunoprecipitation (IP), the entire lanes from the SDS gels were analyzed using mass spectrometry. ND, SlREC2 was not detected. ^a^The accession number is for the UniProt database.

The strategy of first partially purifying multisubunit complexes containing POIs (e.g., using precipitation methods, by purifying organelles, or using some type of chromatography) and ultimately purifying multisubunit complexes containing POIs using an immunoaffinity purification was used to successfully identify the subunits of multisubunit protein complexes in Arabidopsis, albeit infrequently. For example, this approach was used to demonstrate that GUN4, a protein that had no known biochemical function, bound a Mg-chelatase subunit in chloroplast membranes isolated from Arabidopsis. This finding led to experiments demonstrating that GUN4 promotes chlorophyll biosynthesis in Arabidopsis by activating Mg-chelatase (Larkin et al., 2003). This approach was also used to define the subunit composition of Mediator in Arabidopsis. Mediator contributes to the regulation of transcription in diverse eukaryotes (Bäckström et al., 2007). This method has been more commonly used to study multisubunit complexes containing POIs in nonplant systems. Examples that utilized either polyclonal or monoclonal antibodies to ultimately purify multisubunit protein complexes from nonplant systems include the purification of the multisubunit general transcription factors TFIID and TFIIH from metazoans, RNA polymerase II (a multisubunit enzyme) from different eukaryotes, a multisubunit protein complex required for the methylation of histone H3 at lysine residue 4 in *Saccharomyces cerevisiae*, DNA polymerase δ (a two-subunit enzyme) from calf thymus, and vesicular stomatitis virus RNA polymerase (a multisubunit enzyme) from vesicular stomatitis virus-infected baby hamster kidney cells (Dynlacht et al., 1991; LeRoy et al., 1998; Burgess and Thompson, 2002; Qanungo et al., 2004). These methods are suitable for purifying soluble proteins (Bäckström et al., 2007) and proteins from solubilized membranes (Larkin et al., 2003) and can lead to success even if the POI partially dissociates from a multisubunit protein complex during purification and therefore copurifies with the POI in substoichiometric amounts (Larkin et al., 2003). Developing polyclonal antisera against full-length proteins can guard against technical problems, such as protein-protein interactions obscuring the epitopes recognized by anti-POI antibodies. However, a protein completely buried by other proteins in a multisubunit protein complex may not be possible to purify using these methods and may also be difficult to purify using tagging approaches.

We were able to separate SlREC2 from the other SlREC proteins, at least in part because we developed antibodies that specifically bind the nonconserved carboxy terminus of SlREC2. A similar strategy was used to develop antibodies that specifically bind REC1 (Larkin et al., 2016). Distinct strategies may be required to separate proteins that share more sequence similarity than the REC proteins. Specific antibodies may be developed against the most divergent region of a POI even if this region shares sequence similarity with related proteins. Indeed, monoclonal antibodies have been developed that distinguish between proteins encoded by wild-type genes and missense alleles (Trier and Houen, 2023). Polyclonal anti-POI antibodies that cross-react with similar proteins may be removed using affinity chromatography (Nagai et al., 2008; Sabatier et al., 2011). If developing specific antibodies is not possible, even highly similar proteins may fractionate differently on particular resins. A small number of differences in charged or hydrophobic amino acid residues or amino acid residue substitutions in the ligand-binding sites of highly similar proteins may lead to distinct chromatographic behavior on ion exchange, hydrophobic interaction, or affinity columns. Proteins that fractionate similarly on a variety of resins may be better separated using a high-performance liquid chromatography (HPLC) system, which provides better resolution than the gravity-flow and low-pressure chromatography experiments described here. However, gravity-flow and low-pressure chromatography may accommodate larger sample sizes than typical HPLC columns (Jadaun et al., 2016). A POI that elutes from a particular column as a peak that overlaps with a similar protein may be further purified by repeatedly fractionating the POI peak on the same column. Phosphorylated and unphosphorylated forms of a POI may be separated using the Phos-tag linked to agarose (Kinoshita et al., 2005). Different types of glycosylated POIs may be identified by eluting glycosylated POIs from lectin resins using different types of glycosylases or by using specialized columns that bind specific types of glycolytic linkages (Yang and Hancock, 2005; Tian et al., 2007).

Our procedure for purifying SlREC2 illustrates solutions for several common technical problems, such as *in vitro* proteolysis. When *in vitro* proteolysis is an intractable problem, switching experimental systems may lead to success. Thus, we chose to use leaflets as a source of native SlREC2 instead of pericarps. Another solution to this problem is to purify the protein of interest from embryos because embryos are quiescent and therefore contain much lower levels of proteases than metabolically active plant organs. Moreover, embryos that have not been heat treated are commercially available in large quantities for particular species (e.g., wheat germ). We demonstrate only that this method is useful for purifying a native protein from tomato leaflets. Similar strategies were used to purify native proteins from Arabidopsis plants (Larkin et al., 2003; Bäckström et al., 2007) and wheat germ (Thompson et al., 1990). Nonetheless, this method may not be suitable for the purification of native proteins from all plant species and organs.

One critical parameter is that the conditions used to elute the POI from immunoaffinity columns or immunoprecipitates should be identical to the conditions used to elute anti-POI antibodies from the antigen column during the affinity purification of the anti-POI antibodies. Using identical conditions for these elutions ensures that the POI, and therefore, POI-BPs are effectively eluted from the immunoaffinity column (Harlow and Lane, 1999). Suspending the immunoaffinity resin in SDS-PAGE loading buffer and incubating at 100 °C for 5 min will also ensure the effective elution of all proteins, including the POI and POI-BPs, but will also contaminate the eluate with immunoglobulin subunits, which may be unacceptable in some instances. Although a common and effective elution buffer is 100 mM glycine, pH 2.5, there are a variety of other options, such as elution buffers containing organic solvents, chaotropic salts, and denaturing agents. The optimal eluant depends on the nature of the interactions between the antigen and the particular preparation of polyclonal antibodies (Harlow and Lane, 1999). The elution buffer will affect the subsequent analysis. If, for example, only 100 mM glycine, pH 2.5, is used to elute antibodies from antigen columns during the affinity purification and, therefore, to also elute POIs and associated POI-BPs from immunoaffinity columns, prior to analysis using SDS-PAGE, if the POI and POI-BPs in the eluted fractions are too dilute, the eluted fractions can be lyophilized, and the POI and POI-BPs can be resuspended in a smaller volume prior to analysis using SDS-PAGE. Although the combination of 100 mM glycine, pH 2.5, and 50% ethylene glycol may be more effective for eluting antibodies from antigen columns than using only 100 mM glycine, pH 2.5, ethylene glycol cannot be removed using lyophilization. When using an elution buffer that precludes lyophilization, if the POI and POI-BPs in the eluted fractions are too dilute, large volumes of the eluted fractions can be repeatedly loaded onto SDS gels (Sheen and Ali-Khan, 2005), rather than lyophilizing the eluted fractions prior to SDS-PAGE.

The amount of starting material is dependent on the abundance and stability of the POI and is usually established empirically. We obtained sufficient amounts of SlREC2 to readily detect native SlREC2 in Coomassie blue-stained SDS gels if we used 120 g of tomato leaflets and a procedure that included immunoaffinity chromatography. We also obtained sufficient amounts of SlREC2 to readily detect native SlREC2 in Coomassie blue-stained SDS gels if we used much less starting material (i.e., 10 g of tomato leaflets) and a procedure that included immunoprecipitation, although we obtained fewer unique peptides and less sequence coverage from our mass spectrometry analysis when we used less starting material and immunoprecipitation. Immunoaffinity chromatography is usually more effective at purifying large quantities of POIs and quantitatively immunodepleting POIs from solution (compare **Figures 3B, C**; **Supplementary Figure 7**) perhaps because using more immunoaffinity resin and, therefore, a smaller ratio of starting material to affinity-purified anti-POI antibody, is more practical with immunoaffinity chromatography than immunoprecipitation. Another explanation for the more effective immunodepletion of native SlREC2 from chromatographic fractions using immunoaffinity chromatography compared to immunoprecipitation is that the native SlREC2 encountered high local concentrations of the affinity-purified anti-SlREC2 Δ1–1463 antibodies as the native SlREC2 slowly flowed through the immunoaffinity column. Indeed, if only low-affinity antibodies are available, quantitative immunodepletion may be impractical using immunoprecipitation (Harlow and Lane, 1999). The slow percolation of fractions through immunoaffinity columns may produce higher yields of POIs and POI-BPs when only low-affinity antibodies are available. Thus, immunoaffinity chromatography may help to overcome the problem of insufficient yield that may be encountered when using purification procedures involving immunoprecipitation. One strategy for distinguishing contaminants from POI-BPs is to compare data obtained from wild-type plants that accumulate the POI and mutants that do not accumulate the POI. However, mutant alleles, especially mutant alleles that lead to pleiotropic phenotypes, may influence the abundance or even the presence of contaminants and therefore yield misleading data. Distinct strategies for distinguishing contaminants from POI-BPs include fractionating the POI-containing fraction on protein A beads prior to immunoaffinity purification to remove nonspecific-binding proteins and comparing data obtained from purifications conducted using distinct types of ion exchange or affinity chromatography prior to immunoaffinity purification.

If the POI and the POI-BPs copurify in stoichiometric amounts and are present at higher levels than contaminating proteins in the final immunopurified fractions, the identification of POI-BPs using mass spectrometry (Mann et al., 2001) should be straightforward. If the POI and the POI-BPs are not present at substantially higher levels than contaminating proteins, adding additional chromatographic steps prior to the immunoaffinity purification step may help to solve this problem. These procedures can be quantified using a purification summary table, which may help to identify problematic steps. To construct purification tables, POIs are typically quantified using enzyme activity (Burgess, 2009). When an enzyme assay is not available, we recommend quantifying immunoreactive bands on immunoblots. Using this method, we found that a major loss of SlREC2 occurred during the ultrafiltration and gel filtration steps of the procedure that utilized immunoaffinity chromatography (**Table 1**). After eliminating these steps, we obtained a much higher yield from a procedure that utilized immunoprecipitation (**Table 3**), even though immunoaffinity chromatography was more effective at immunodepleting SlREC2 than immunoprecipitation (**Figure 3**; **Table 1**, **Table 3**). Perhaps boiling the immunoprecipitates in SDS-PAGE loading buffer more effectively eluted SlREC2 than 100 mM glycine, pH 2.5, 50% ethylene glycol at 4 °C. Nonetheless, we were able to obtain quantities of SlREC2 that we could detect in Coomassie blue-stained SDS gels using both procedures (**Figures 4A, B**).

**Table 3:** Summary of SlREC2 purification using immunoprecipitation.

Step^a^	Concentration (mg/ml)^b^	Volume (ml)	Protein (mg)	Gray value (units)^c^	Total SlREC2 (units)^d^	SlREC2 purity (units/mg)^e^	Fold Purification^f^	Yield (%)^g^
Supernatant	3.51	14	49.1	7190	101000	2060	1	100
SP Sepharose	0.27	4	1.1	9890	39600	36000	18	39
Q Sepharose	0.14	0.3	0.042	15900	4770	113000	55	4.7
Immunoprecipitation		0.0475	0.012	51600	2450	204000	100	2.4

a Pertinent data is indicated for each fraction produced during the purification of SlREC2 using immunoprecipitation. Values for each purification step were obtained from pooled fractions containing SlREC2.

b Protein concentrations were determined using the Bradford assay with BSA serving as the standard protein.

c The amount of SlREC2 was quantified from images of immunoblots using ImageJ. Grey value refers to the intensity of the signal from a protein band generated during the quantification procedure.

d Total SlREC2 was calculated by multiplying the gray value by the volume of the pooled fractions.

e SlREC2 purity was calculated by dividing the gray value by the amount of protein in the pooled fractions.

f Fold purification was calculated by dividing SlREC2 purity in a particular pool of fractions by SlREC2 purity in the supernatant.

g Percent yield is the percent of total SlREC2 in a particular pool of fractions relative to the total SlREC2 in the supernatant.

Physiologically meaningful POI-BPs can be further distinguished from contaminants using anti-POI-BP antibodies and extracts prepared from wild-type plants in a variety of experiments. For example, if the POI coimmunoprecipitates with anti-candidate POI-BP antibodies and vice versa or copurifies on immunoaffinity columns constructed using the anti-candidate POI-BP antibodies and vice versa, such data demonstrates that the POI and the POI-BP stably associate in plant extracts and is compelling evidence that they stably associate *in vivo*. Other evidence that the POIs and POI-BPs interact can come from coelution from gel filtration columns, cosedimentation in density gradients, and comigration in native gels. Experiments involving crosslinking followed by SDS-PAGE can also demonstrate that POIs and POI-BPs interact (Tang and Bruce, 2009). Experiments using heterologous systems, such as two-hybrid methods (Vidal and Fields, 2014) and high-level expression in *Nicotiana benthamiana*, including biomolecular fluorescence complementation assays, luciferase complementation assays (Bais et al., 2023), and co-immunoprecipitation (Nam et al., 2023), provide evidence the POI and POI-BP have the capacity to interact. Phenotypic characterizations of mutants deficient in the POI and mutants deficient in the candidate POI-BPs can also provide evidence that particular POI-BPs are biologically meaningful.

The purification of native POIs from wild-type plants can be achieved rapidly. For the purification of SlREC2 ultimately using immunoaffinity chromatography, the different steps of the procedure, including homogenization, ion exchange chromatography, ultrafiltration, and loading onto a preparative gel filtration column, were completed in one day. Preparative gel filtration chromatography was performed overnight. All the fractions were analyzed by immunoblotting or dot blotting on the second day. The immunoaffinity chromatography was done on the third day and was analyzed subsequently. For the purification of SlREC2 ultimately using immunoprecipitation, the different steps of the procedure, including homogenization, ion exchange chromatography, and immunoprecipitation, were done in one day. All the fractions were analyzed by immunoblotting on the second day. Gravity flow chromatography and step gradients require no expensive equipment, although higher-resolution purification experiments can be performed using an automated chromatography system.

## 4 Conclusions

The impetus for this report was to provide a rationale and guidance for purifying native proteins from wild-type plants, an underutilized approach in plant systems. We provide advice on method development and on troubleshooting, and explain the advantages of partial purification prior to immunoaffinity purification relative to procedures that immunoprecipitate POIs directly from whole-organ extracts. As an illustrative example, we developed procedures for the rapid purification of native SlREC2 from tomato leaflets using either immunoaffinity chromatography or immunoprecipitation with affinity-purified anti-SlREC2 antibodies. Our best procedure provided a 100-fold purification of SlREC2 with a 2.4% yield. The yield may have been increased by using immunoaffinity chromatography, which appeared to more effectively immunodeplete SlREC2 from column fractions than immunoprecipitation. Although our procedures provided only low yields of SlREC2, sufficient quantities of SlREC2 were obtained to allow for the detection of SlREC2 in SDS gels stained with Coomassie blue and to unambiguously detect SlREC2 using mass spectrometry. Our procedures demonstrate how to overcome excessive proteolysis using rapid batch-binding and elution procedures that allow for the rapid capture and partial purification of native proteins prior to immunoaffinity purification and how to improve yield by trying different resins and buffers. Experimenting with different salts, glycerol, and detergents may also improve yield.

## Data Availability

Unprocessed immunoblots and Coomassie-stained SDS gels are provided.
