# Supplementary material for: Purification of SlREC2 from wild-type tomato leaflets using immunoaffinity chromatography and immunoprecipitation
Source: Front Plant Sci. 2025 Dec 17;16:1616665. doi: 10.3389/fpls.2025.1616665 (PMC12753505; doi:10.3389/fpls.2025.1616665)
Supplement: Supplementary file 1 [file DataSheet1.pdf]

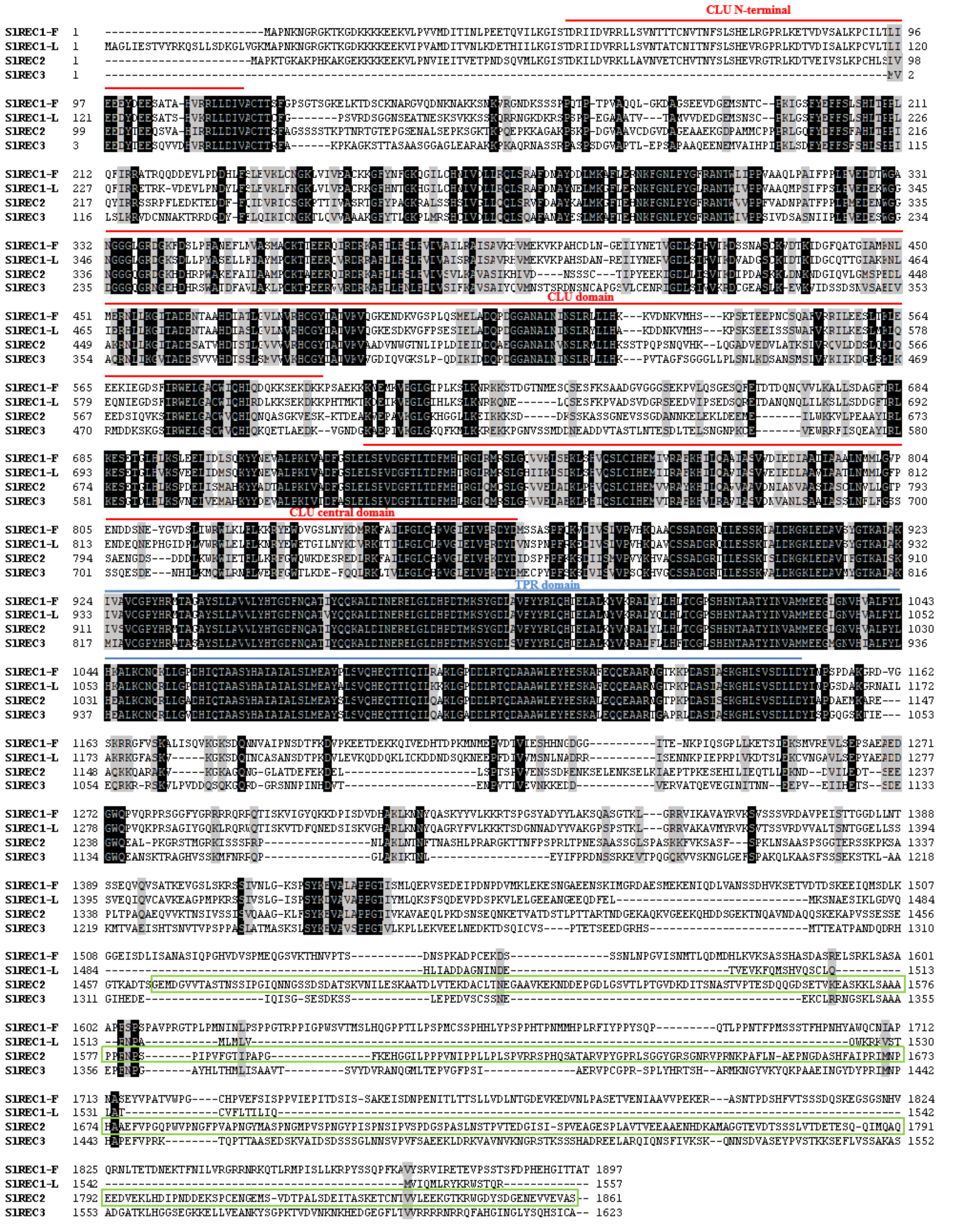

Supplementary Figure 1. Amino acid sequence alignment and domain analysis of SlREC proteins from tomato. Identical amino acids are shaded with black. Similar amino acids are shaded with gray. The conserved CLU and TPR domains are indicated with red lines and blue lines, respectively. The fragment of SlREC2 used to prepare anti-SlREC2 antibodies is indicated with green boxes.

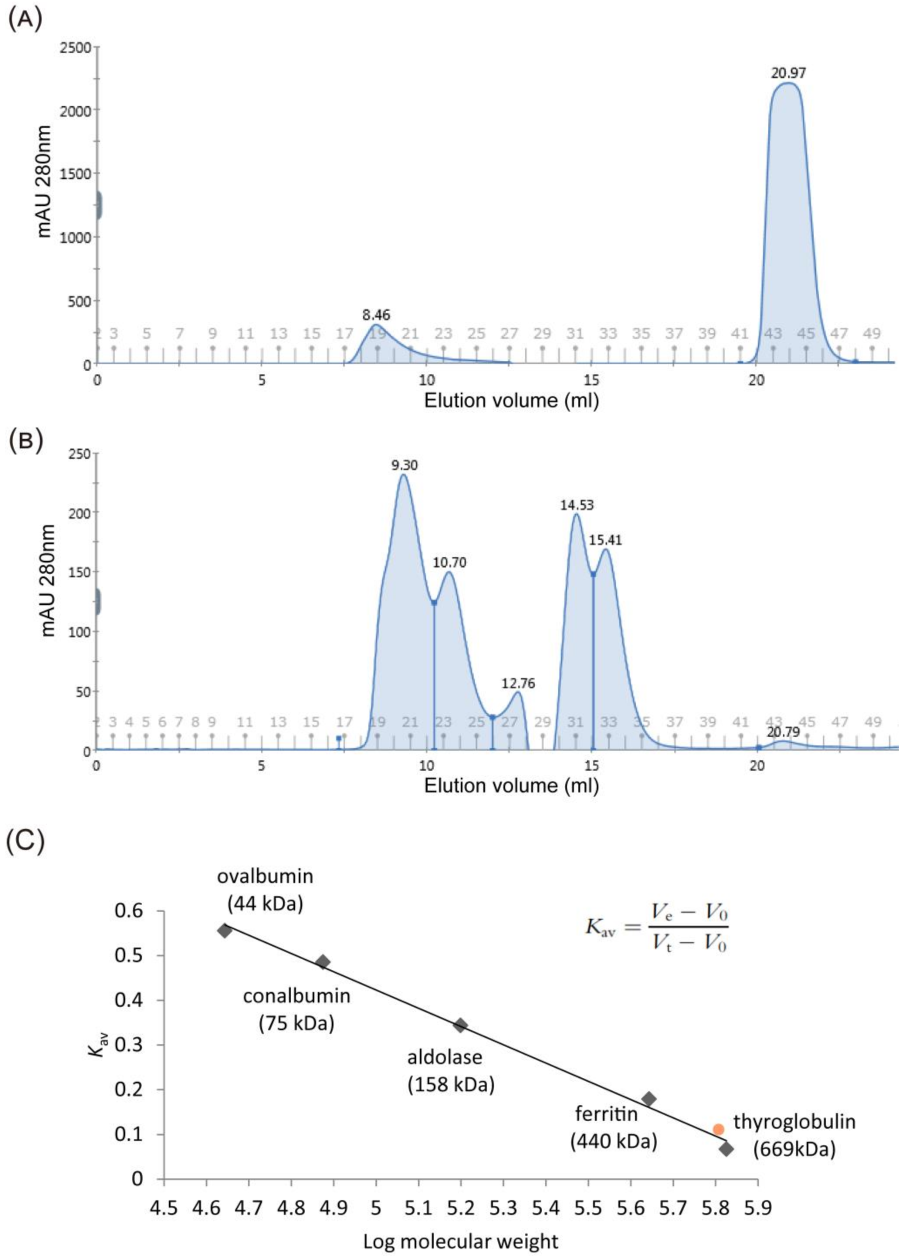

Supplementary Figure 2. Mass estimates for native SlREC2 extracted from leaflets and fruit. (A). Chromatograms for blue dextran and vitamin B_{12}. Blue dextran and vitamin B_{12} were fractionated on a Superdex 200 Increase 10/300 GL column. The elution peak for blue dextran at 8.46 mL was defined as the void volume (V_0). The elution peak for vitamin B_{12} at 20.97 mL was defined as the total volume (V_t).

(B) Chromatograms for standard proteins. Standard proteins were fractionated on a Superdex 200 Increase 10/300 GL column. The peak volumes in mL are indicated above each peak. The standard proteins were thyroglobulin (669 kDa), ferritin (440 kDa), aldolase (158 kDa), conalbumin (75 kDa), ovalbumin (44 kDa).

(C) Plot of K_{av} vs log molecular weight. K_{av} was correlated with log molecular weight, $R^2 = 0.99$. The standard proteins are indicated with grey diamonds. The orange circle represents the SlREC2 protein extracted from tomato fruits and leaflets.

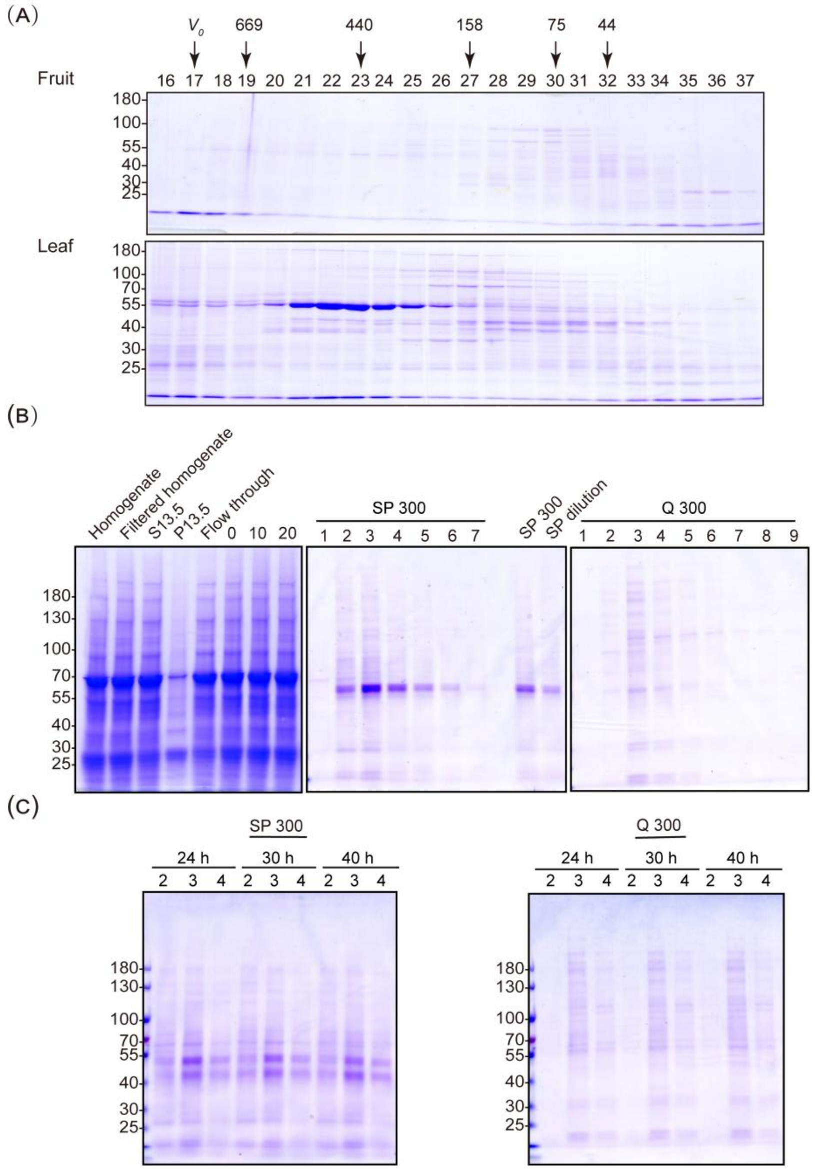

Supplementary Figure 3. Total protein in extracts and fractions containing SlREC2.

(A) Elution of pericarp and leaflet protein from a gel filtration column. Whole-fruit pericarp extracts (top) from B+2 fruit and 30-d-old whole-leaflet extracts (bottom) were fractionated on a Superdex 200 Increase 10/300 GL column. Equal volumes of each fraction were analyzed using 10% SDS gels stained with Coomassie blue R-250. The void volume (V_0) is indicated. The fractions containing the elution peaks for each standard protein are indicated with the mass of the pertinent standard protein in kDa. The relative mobilities of molecular weight standard proteins for the SDS gels are indicated at the left in kDa.

(B) Binding and elution of leaflet protein from SP Sepharose and Q Sepharose. Crude 30-d-old leaflet extracts (Homogenate) were filtered through Miracloth (Filtered homogenate) and clarified by centrifugation at 13,500 × *g* to yield supernatant (S13.5) and pellet (P13.5) fractions. Equal volumes of these fractions, the unbound (Flow through), and fractions eluted from SP Sepharose and Q Sepharose using 300 mM NaCl (SP 300 and Q 300), and the SP300 diluted with buffer H (SP dilution) were analyzed using 8% SDS gels and stained with Coomassie blue R-250. The amount of protein remaining after the S13.5 was incubated at

4 °C for 0, 10, and 20 min is shown.

(C) Protein in fractions eluted from SP Sepharose and Q Sepharose. Protein was purified from leaflets using SP Sepharose and Q Sepharose as described in **(B)**.The amount of protein remaining after three fractions from the elution peaks (fractions 2, 3 and 4) from SP Sepharose and Q Sepharose were incubated at 4°C for 24, 30 and 40 h is shown. Equal volumes of each fraction at each time point were analyzed using 8% SDS gels stained with Coomassie blue R-250. The relative mobilities of molecular weight standard proteins are indicated at the left in kDa.

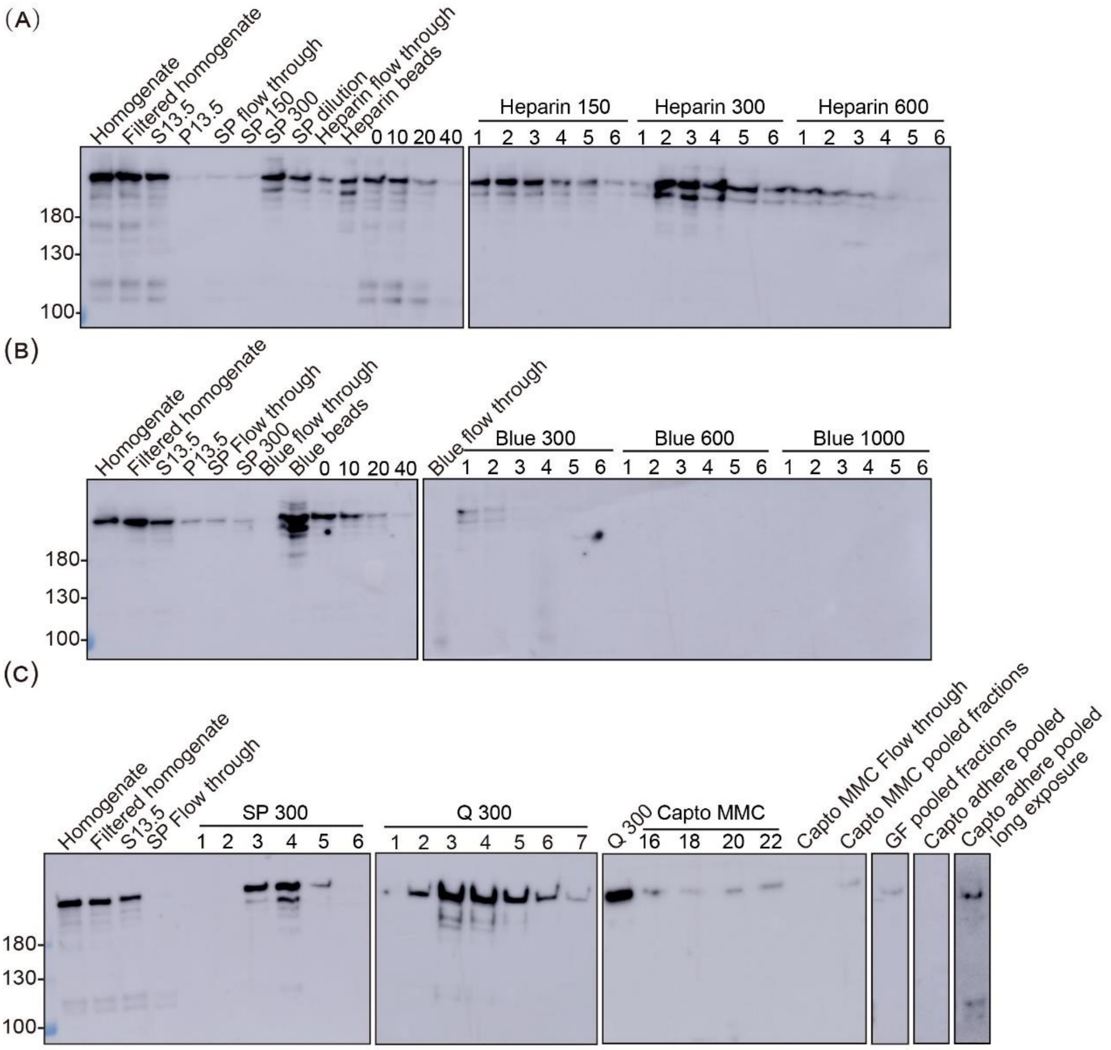

Supplementary Figure 4. Chromatographic analysis of SlREC2.

(A) Binding and elution of SlREC2 from SP Sepharose and Heparin Sepharose. Whole 32-d-old leaflet extracts (Homogenate) were filtered through Miracloth (Filtered homogenate) and clarified by centrifugation at 13,500 × *g* to yield supernatant (S13.5) and pellet (P13.5) fractions. The S13.5 was batch bound to SP Sepharose yielding a fraction that flowed through the SP Sepharose column (SP flow through) and fractions produced by washing the SP Sepharose column with buffer F (SP 150) and eluting the SP Sepharose column with buffer G (SP 300). The SP300 fraction was diluted with an equal volume of buffer H (SP dilution) and batch bound to Heparin Sepharose yielding a fraction that flowed through the Heparin Sepharose column (Heparin flow through). Heparin Sepharose beads (5 μL) after elution with

buffer G and I, and the S13.5 incubated at 4 ℃ for 0, 10, 20, and 40 min were also analyzed (left). Equal volumes of these fractions were analyzed using 8% SDS gels and immunoblotting with affinity-purified anti-SlREC2 Δ1-1463 antibodies (left). Heparin Sepharose was washed using buffer F (Heparin 150) and then eluted with buffer G (Heparin 300) and then with buffer I (Heparin 600) (right). Equal volumes of these fractions were analyzed using 8% SDS gels and immunoblotting with affinity-purified anti-SlREC2 Δ1-1463 antibodies.

(B) Binding and elution of SlREC2 from SP Sepharose and Blue Sepharose. Whole 48-d-old leaflet extracts were fractionated on SP Sepharose as described in (A) (left). The SP 300 fraction was diluted with buffer H to a final salt concentration of 100 mM and fractionated on Blue Sepharose 6 Fast Flow essentially as described for Heparin Sepharose in (A) except that after eluting the Blue Sepharose column with buffer I, the blue Sepharose was eluted with buffer K. Equal volumes of the different fractions were analyzed using immunoblotting as described in (A).

(C) Binding and elution of SlREC2 from SP Sepharose, Q Sepharose, Capto MMC, a gel filtration column, and Capto adhere. Whole 38-d-old leaflet extracts were fractionated on SP Sepharose as described in (A). The SP300 fraction was diluted with an equal volume of buffer H, batch bound to Q Sepharose and eluted from Q Sepharose using buffer G. The fractions that were eluted from Q Sepharose were pooled (Q 300) and fractionated on a HiTrap Capto MMC column. The eluted fractions that contained SlREC2 (Capto MMC) were pooled (Capto MMC pooled fractions) and applied to a HiPrep 26/60 Sephacryl S-300 High Resolution gel filtration column. The fractions that eluted from the gel filtration column containing SlREC2 were pooled (GF pooled fractions) were applied to a HiTrap Capto adhere column. SlREC2 was not detectable in the fractions that eluted from the HiTrap Capto adhere column (Capto adhere pooled) without an extended exposure of the immunoblot (Capto adhere pooled long exposure). Equal volumes of the different fractions were analyzed using immunoblotting as described in (A).

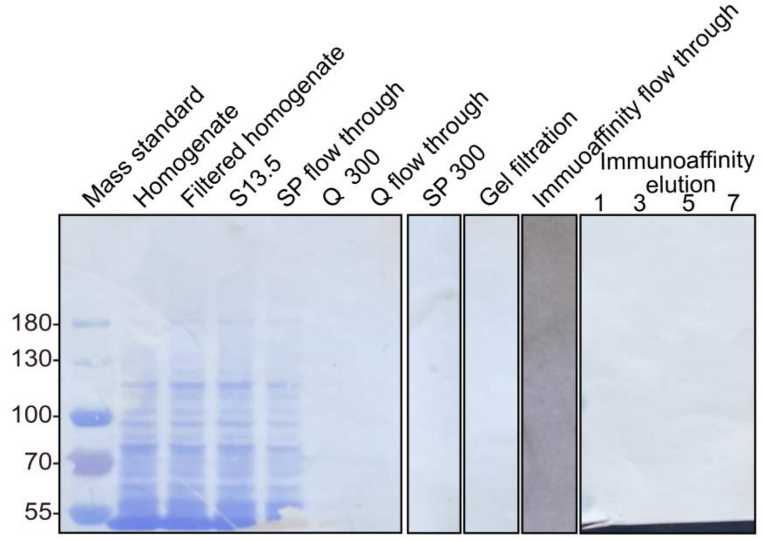

Supplementary Figure 5. Total protein in key fractions from the purification of SlREC2 from leaflets ultimately using immunoaffinity chromatography. Whole-leaflet extracts prepared from 38-d-old leaflets (Homogenate) were filtered through Miracloth (Filtered

homogenate) and clarified by centrifugation at 13,500 × g to yield a supernatant (S13.5). Fractions containing SlREC2 and flow-through fractions from the indicated steps are shown. SP 300 and Q 300 refer to fractions eluted from SP Sepharose and Q Sepharose, respectively, using a buffer containing 300 mM NaCl. Equal volumes of each fraction were analyzed using 6.5% SDS gels and immunoblotting with affinity-purified anti-SlREC2 Δ1-1463 antibodies. After the immunoblotting procedure, the PVDF membranes were stained with Coomassie brilliant blue. The relative mobilities of the molecular weight standards are indicated at the left in kDa.

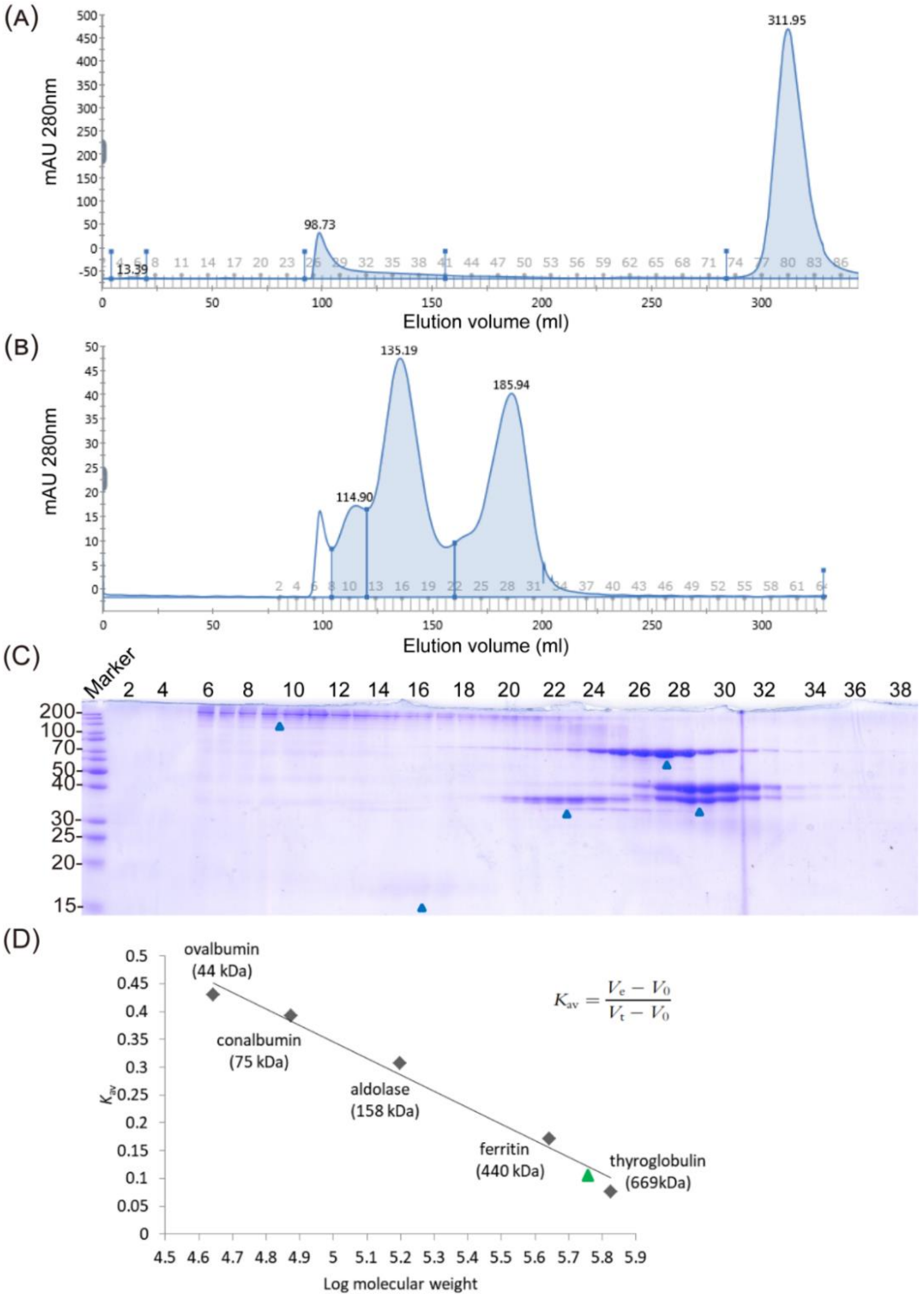

Supplementary Figure 6. Mass estimate for native SlREC2 purified from leaflets.

(A). Chromatograms for blue dextran and vitamin B_{12}. Blue dextran and vitamin B_{12} were fractionated on a HiPrep 26/60 Sephacryl S-300 High Resolution gel filtration column. The

elution peak for blue dextran at 98.73 mL was defined as the void volume (V_0). The elution peak for vitamin B_{12} at 311.95 mL was defined as the total volume (V_t).
(B) Chromatograms for standard proteins. Standard proteins were fractionated on a HiPrep 26/60 Sephacryl S-300 High Resolution gel filtration column. The unshaded peak indicates the V_0. The peak fractions for two of the standard proteins were at 114.9mL (thyroglobulin, 669 kDa) and 135.19 mL (ferritin, 440 kDa). Aldolase (158 kDa), conalbumin (75 kDa) and ovalbumin (44 kDa) were not clearly resolved based on absorbance at 280 nm used by the in-line UV detector and eluted with a peak fraction at 185.94 mL.
(C) Elution profiles of standard proteins. Equal volumes of fractions containing standard proteins described in (B) were analyzed using a 12% SDS gel and stained with Coomassie blue R-250. The blue triangles indicate the peak fractions for each standard protein. The peak volumes were 115 mL (thyroglobulin, 669 kDa), 135 mL (ferritin, 440 kDa), 164 mL (aldolase, 158 kDa), 182 mL (conalbumin, 75 kDa), and 190 mL (ovalbumin, 44 kDa). The mass of each molecular weight marker protein for the SDS gel is indicated at the left in kDa.
(D) Plot of K_{av} vs log molecular weight. K_{av} was correlated with log molecular weight, $R^2 = 0.98$. The standard proteins are indicated with grey diamonds. The green triangle represents the SlREC2 protein purified from tomato leaflets.

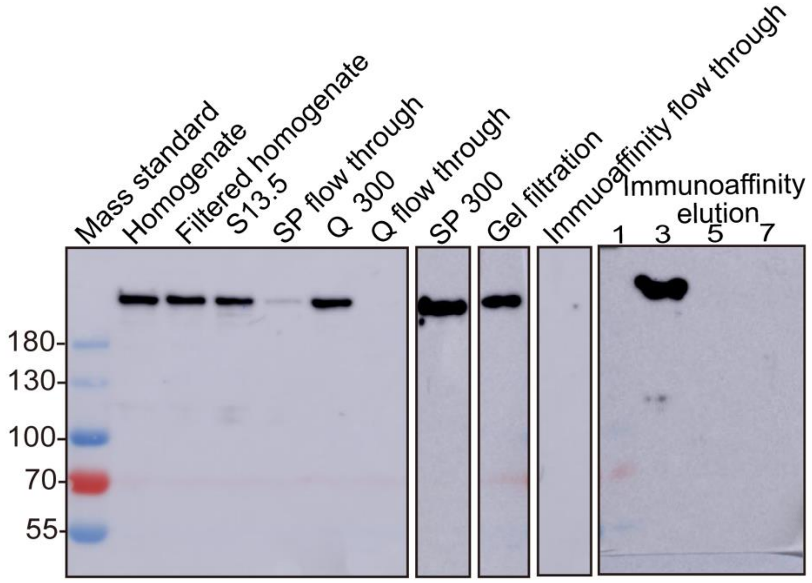

Supplementary Figure 7. Extended exposure of key fractions from the purification of SlREC2 from leaflets ultimately using immunoaffinity chromatography. Whole-leaflet extracts prepared from 38-d-old leaflets (Homogenate) were filtered through Miracloth (Filtered homogenate) and clarified by centrifugation at 13,500 × g to yield a supernatant (S13.5). Fractions containing SlREC2 and flow-through fractions from the indicated steps are shown. SP 300 and Q 300 refer to fractions eluted from SP Sepharose and Q Sepharose, respectively, using a buffer containing 300 mM NaCl. Equal volumes of each fraction were analyzed using 6.5% SDS gels and immunoblotting with affinity-purified anti-SlREC2 Δ1-1463 antibodies. The immunoblots for the SP 300, gel filtration, immunoaffinity flow through and immunoaffinity elution fractions were exposed separately from the immunoblot of the homogenate and other fractions.
